# Pairing Red Wine and Closure: New Achievements from Short-to-Medium Storage Time Assays

**DOI:** 10.3390/foods14050783

**Published:** 2025-02-25

**Authors:** João Mota, André Viana, Cátia Martins, Adriana C. S. Pais, Sónia A. O. Santos, Armando J. D. Silvestre, José Pedro Machado, Sílvia M. Rocha

**Affiliations:** 1 LAQV-REQUIMTE & Department of Chemistry, University of Aveiro, Campus Universitário Santiago, 3810-193 Aveiro, Portugal; joao.mota21@ua.pt (J.M.); apviana@ua.pt (A.V.); catiamartins@ua.pt (C.M.); 2 CICECO-Aveiro Institute of Materials & Department of Chemistry, University of Aveiro, Campus Universitário Santiago, 3810-193 Aveiro, Portugal; a.c.p.s@ua.pt (A.C.S.P.); santos.sonia@ua.pt (S.A.O.S.); armsil@ua.pt (A.J.D.S.); 3 M.A.Silva Cortiças S.A., Rua Central das Regadas Nº49, 4535-167 Mozelos, Portugal; jpmachado@masilva.pt

**Keywords:** cork stoppers and alternatives, wine post-bottling, wine storage, phenolic compounds, volatile compounds, chromatography

## Abstract

The physicochemical and sensory properties of wines are influenced by several factors, starting in the vineyard and evolving during the winemaking stages. After bottling, variables such as bottle position, closure type, storage temperature, and storage time shape wine characteristics. In this study, red wines stored for approximately 0.5 and 3 years with natural cork, micro-agglomerated cork stoppers, and screw cap closures were analyzed. Various techniques were employed to investigate changes during bottle storage, including the determination of volatile components by comprehensive gas chromatography-mass spectrometry with time-of-flight analyzer (GC × GC-ToFMS), phenolic profile by ultra-high-performance liquid chromatography, coupled with tandem mass spectrometry (UHPLC-DAD-MS^n^), general physicochemical parameters, the oxygen transfer rate of cork stoppers, and sensorial analysis performed by a trained panel. The results revealed that the type of closure created distinct environments within the bottles, slightly influencing both sensory attributes and chemical evolution of the red wines. These findings highlight the value of combining diverse analytical techniques to reveal closure-driven differences, with volatile compound profiling emerging as the most sensitive methodology. Additionally, this study emphasizes that differences modulated by the wine–closure pairing, which become more pronounced during storage, can serve as an oenological tool in the construction of a wine’s identity.

## 1. Introduction

Wine is a relevant beverage across many regions of the world [1], and its sensory characteristics have been the focus of extensive research over time. Sensory attributes are pivotal for determining consumer acceptance, assessing quality, and ensuring authenticity. The study of wine’s sensory and physicochemical properties has gained interest in recent years, driven by growing interest from researchers and companies who wish to explore, improve, and adapt wines to align with market trends and innovation demands. The unique physicochemical and sensory profile of wines emerges from a complex interplay of factors, beginning in the vineyard and extending through every stage of winemaking. After bottling, the wine undergoes a period of storage under varying conditions that can significantly influence its characteristics. During this phase, several factors play a role, including the size and material of the closure, the dimensions of the bottleneck and closure, the storage temperature and duration, and the storage positioning of the bottle, among others [2]. Red wines, in particular, undergo notable changes during storage, and their quality is often expected to improve over time. The evolution of red wines is primarily driven by transformations in their phenolic composition, which affect their color, mouthfeel, and oxidation levels. Additionally, wine’s volatile composition evolves depending on several factors, with exposure to oxygen being a critical factor. While controlled oxygen levels can contribute positively to the development of aromas and complexity, excessive oxygen can lead to oxidative spoilage [3]. The permeability of closures to oxygen levels plays an important role in modulating wine’s sensory properties during storage, influencing its evolution and overall quality [1,4], with high oxygen transfer rate (OTR) levels accelerating oxidative processes, and low-OTR closures, such as screw caps, creating a reductive environment [5,6]. Moreover, successful headspace oxygen management at and post-bottling is possible when in-depth knowledge of the impact of bottling and storage on oxygen levels and wine quality is achieved [7]. Most modern bottling lines can achieve displacement of approximately 60–80% of the oxygen from the headspace, resulting in reasonably low headspace oxygen values of around 0.8–1.5 mg/L [8].

There are several types of closures commonly used in winemaking, including natural and micro-agglomerated cork stoppers, and screw cap closures, which represent the focus of the present study. Natural cork stoppers, sourced from the cork oak bark (*Quercus suber* L.), allow moderate oxygen ingress, supporting gradual maturation of the wine and the development of complex sensory attributes [1,4]. Micro-agglomerated cork stoppers, made from granulated cork particles bonded with food-grade resins, provide a more uniform structure than natural cork [4,9]. Screw caps, which are typically made of aluminum with an inert liner, create an almost airtight seal, effectively minimizing oxygen ingress, but their prolonged use can sometimes result in reductive conditions, altering the wine’s aroma and flavor over time [1,4,10].

Previous studies suggest that the type of closure used might play a key role in preserving the quality, shelf life, and evolution of wines, with volatile organic and phenolic compounds being of particular importance [1,11]. Natural cork stoppers have been shown to better preserve ethyl esters—compounds associated with fruity and sweet aromas—especially over long storage periods. These notes are commonly appreciated by consumers [12,13,14,15]. Conversely, the presence of high amounts of sulfur compounds has been reported more frequently in wines sealed with screw caps, which may explain the reductive aromas (off-flavors) [16,17,18]. Some studies, however, suggest that aroma perception is not significantly influenced by the type of closure used [19,20]. Regarding phenolic compounds, during in-bottle storage, in addition to migration phenomena from cork stoppers, oxidation–reduction reactions may also occur depending on the type of closure used [11,21]. Oxidative polymerization reactions, which can cause a rapid decline in free anthocyanins in wines in the presence of dissolved oxygen, have been assigned specifically to natural cork stoppers [11].

While these studies have provided valuable insights into specific aspects of closures and their impact on wines’ chemical and sensorial characteristics, none of them have conducted an extensive and holistic evaluation that encompasses multiple critical parameters of wines, such as their general physicochemical characterization, sensory profile, and volatile and phenolic composition. Furthermore, research on the impact of closures on red wines remains limited, as most studies have focused on white and sparkling wines [2,12,13,22]. Therefore, this study aims to address this gap and was designed to holistically evaluate the impact of different types of closures on two blended red wines from different Appellations, with short and medium in-bottle storage periods: one from the Douro Appellation (Portugal), and the other from Burgenland (Austria). The study employed natural and micro-agglomerated cork stoppers and screw cap closures. To achieve the objective, a comprehensive set of analyses was implemented, including the determination of volatile compounds using advanced gas chromatography techniques, such as comprehensive gas chromatography–mass spectrometry with a time-of-flight analyzer (GC × GC-ToFMS), and the determination of phenolic profiles through ultra-high-performance liquid chromatography coupled with tandem mass spectrometry (UHPLC-DAD-MS^n^). Additional analyses covered OTR, chromatic parameters, acidity (total and volatile), sulfur dioxide levels (free and total), pH, and sensorial evaluations conducted by experienced panels. Data from the various analytical domains were processed and integrated using univariate and multivariate statistical tools to draw meaningful conclusions. The wine profiles explored in this study highlight how the interplay between closure and in-bottle storage duration influences the chemical and sensory pathways of red wines, providing a more integrated understanding of their behavior during short-to-medium-length storage periods.

## 2. Materials and Methods

### 2.1. Materials and Reagents

Cyanidin chloride (purity higher than 98%), gallic acid (purity higher than 97.5%), quercetin (purity higher than 98%), Trolox, and 2,2-diphenyl-1-picryl-hydrazyl (DPPH) were supplied by Sigma Chemical Co. (Madrid, Spain). 3-Octanol (99% purity) was purchased from Aldrich Chemical Company (Milwaukee, WI, USA). Caffeic acid (purity > 95%), naringenin (98% purity), and vanillin (99% purity) were obtained from Aldrich Chemicals Co (Madrid, Spain). Formic acid (96% purity), Catechin (purity > 96%), and ellagic acid (96% purity) were purchased from Fluka Chemie (Madrid, Spain). HPLC-grade methanol (99.8% purity) and acetonitrile (99.9% purity) were obtained from Fisher Scientific Chemicals (Loures, Portugal). LC-MS-grade water was purchased from Sigma Aldrich Canada Co. (Oakville, ON, Canada). Sodium chloride (99.8% purity) was supplied by José Manuel Gomes dos Santos, LDA (Odivelas, Portugal). The retention index probe (an n-alkanes series of C8 to C20 straight-chain alkanes, in hexane) was obtained from Fluka (Buchs, Switzerland). The solid phase microextraction (SPME) holder for manual sampling and the fiber coating used were acquired from Supelco (Aldrich, Bellefonte, PA, USA), which included a 1 cm StableFlex™ fused silica fiber coated with divinylbenzene/carboxen/poly(dimethylsiloxane) (DVB/CAR/PDMS—50/30 μm). The SPME fibers were conditioned according to the manufacturer’s recommendations.

### 2.2. Red Wines and Closures

A short (5 months) and medium (35 months) in-bottle storage time were selected to gain insight into the impact of storage duration and closure. Two red wines (blends) from two distinct demarcated regions were selected:

- Red wine from the Douro Appellation (sub-region Cima Corgo, São João da Pesqueira, Portugal) produced from a blend of three red grape varieties, Touriga Nacional, Touriga Franca, and Tinta Amarela, with an alcohol content of 15% (*v*/*v*). It was bottled in January 2020 and stored for 35 months until analysis. The bottles were kept in a horizontal position, away from light, at temperatures ranging from 14 °C to 17 °C. For this assay, natural (Natural Cork 1) and micro-agglomerated cork stoppers (Micro A) (Table 1) were used.
- Red wine, produced from a blend of four red grape varieties, Blaufränkisch, Cabernet Sauvignon, Merlot, and Zweigelt, from Burgenland (Mittelburgenland, Austria), with an alcohol content of 14,5% (*v*/*v*). It was bottled in December 2022 and was stored for 5 months until analysis, in a horizontal position, away from light, at temperatures between 14 °C and 16 °C. Four types of closures were used for this wine: natural (Natural Cork 2) and two micro-agglomerated cork stoppers (Micro A and Micro E), and screw cap tin saran closure (Screw Cap) (Table 1).

### 2.3. Determination of Oxygen Transfer Rate of Stoppers

Determination of the oxygen transfer rate (OTR) was performed for natural and micro-agglomerated cork stoppers. Due to technical constraints, at this stage, it was not possible to carry out this determination for screw cap tin saran closures. Transparent bottles with a volume of 750 mL with a standard neck diameter of 18.5 mm and a fill level of 63 mm from the neck entrance were used, which were previously washed with 99.9% (*v*/*v*) ethanol, followed by a rinse with distilled water for approximately 1 min. The bottles were previously saturated with nitrogen and then sealed using CITcork (Egitron, Santa Maria da Feira, Portugal) with a compression diameter of 16 mm and an insertion and compression speed of 1500 cm/min and 240 cm/min, respectively. An initial reading of the partial pressure of oxygen was taken to ensure nitrogen saturation. OTR measurements were carried out using the chemiluminescence method using a fiber-optic device, NomaSense O2 P6000, and Pst6 sensor spots (PreSens Precison Sensing GmbH, Regensburg, Germany). The device emits a beam of infrared light through the fiber to the optical sensor inside the bottles, stimulating it. The amount of oxygen in the sensor is proportional to the delay in the red-light emission generated by the electrons’ return to the ground state. This is expressed as partial pressure of oxygen, which is then converted into milligrams of oxygen by the ideal gas law. In addition, a Pt100 temperature probe (PreSens Precison Sensing GmbH, Germany) was used, which connects to the equipment and allows it to be automatically compensated throughout the various measurements. Five measurements were taken per analysis time and closure type, with results expressed as mg O_2_ (average) ± standard deviation.

### 2.4. Determination of General Physicochemical Properties of Wines

Regarding the determination of general physicochemical properties of wines and to ensure the reliability and accuracy of the results, three independent aliquots of each wine were analyzed per bottle, in a total of four bottles per type of closure (4 bottles × 3 replicates, n = 12).

#### 2.4.1. pH

The pH was measured using a pH meter (micropH 2002, Crison, Barcelona, Spain) previously calibrated with pH 4 and pH 7 buffer solutions.

#### 2.4.2. Total Acidity

The total acidity of the red wines, excluding carbon dioxide, was measured using potentiometric titration according to the OIV-MA-AS313-01 method [23]. For this procedure, approximately 50 mL of wine was placed in a vacuum flask and degassed using a vacuum pump for 1–2 min. The titration was carried out with a pH meter calibrated using a pH 7.0 buffer solution. A 10 mL sample of the degassed wine was titrated with 0.1 mol/L sodium hydroxide while stirring continuously until the pH reached 7 at 20 °C. The volume of sodium hydroxide used was recorded for further calculations. The results were expressed in milliequivalents per liter (meq/L) and converted to grams of tartaric acid per liter (g/L tartaric acid) for comparison.

#### 2.4.3. Volatile Acidity Deduced from SO_2_

The volatile acidity of red wines, which comprises the free and combined acids of the acetic series, was determined using steam distillation followed by potentiometric titration, following OIV-MA-AS313-02 [23]. Initially, carbon dioxide was removed from the wine sample by applying a vacuum to approximately 50 mL of the wine in a vacuum flask. Subsequently, about 20 mL of the treated wine was distilled, along with the addition of approximately 0.5 g of tartaric acid, to enhance the separation of volatile acids. The collected distillate was titrated with a standard sodium hydroxide solution (0.1 M) using phenolphthalein as an indicator. The amount of sodium hydroxide used in the titration was carefully recorded. The results of the titration were then converted to grams per liter of acetic acid, allowing for an accurate assessment of the wine’s volatile acidity, which is essential for evaluating its quality and stability. In addition to the total volatile acidity, the volatile acidity deduced from sulfur dioxide (SO_2_) is also an important parameter. This involves subtracting the acidity contributed by free and combined SO_2_ from the total volatile acidity measured.

#### 2.4.4. SO_2_ Free and Total

The sulfur dioxide levels in wines, encompassing both free and total sulfur dioxide, were evaluated according to the OIV-MA-AS323-04A1 method [23]. Initially, approximately 50 mL of the wine sample was transferred into a flask and treated with 15 mL of diluted phosphoric acid to create an acidic environment for the entrainment process. A flow of air or nitrogen was then introduced into the sample, facilitating the transfer of free sulfur dioxide through a bubbler, where it was oxidized to sulfuric acid by a hydrogen peroxide solution. The formed sulfuric acid was subsequently titrated with a standard 0.01 M sodium hydroxide solution until a neutral pH was achieved; this was indicated by a color change. The volume of sodium hydroxide used in the titration was recorded, and the free sulfur dioxide concentration was calculated in milligrams per liter (mg/L). The total sulfur dioxide levels in wine, representing the sum of free and bound forms, were determined using the OIV-MA-AS323-04A2 method [23]. Approximately 50 mL of wine sample was introduced into a flask, followed by the addition of 15 mL of phosphoric acid to establish an acidic medium conducive to the entrainment process. An airflow was then initiated to transfer the sulfur dioxide through a bubbler, where it was oxidized to sulfuric acid via a hydrogen peroxide solution. The resulting sulfuric acid was titrated with a 0.01 M sodium hydroxide solution until the endpoint was reached, as indicated by a color shift. The volume of sodium hydroxide consumed during titration was noted, allowing for the calculation of the total sulfur dioxide concentration in milligrams per liter (mg/L).

#### 2.4.5. Chromatic Parameters

The chromatic parameters were evaluated using a spectrophotometric method, with absorbance measurements conducted using a UV-vis V-530 spectrophotometer (Jasco, Tokyo, Japan). Absorbance was recorded at wavelengths of 420 nm, 520 nm, and 620 nm. According to the OIV-MA-AS2-07B method [23], the intensity of the color (I) is calculated as the sum of the absorbance values at these three wavelengths, and the shade of the wines is represented by the ratio between the absorbance at 420 nm and at 520 nm. Additionally, the percentages of red, yellow, and blue components were calculated based on the absorbance values at each respective wavelength, according to the method established by Glories [24].

### 2.5. Sensorial Properties for Evaluation of the Oxireduction Level of Wines

For the sensorial analysis of the Douro red wines, a panel of 10 members from a cork company was used, consisting of 6 men and 4 women. The panel was trained to assess oxidation and reduction attributes using a scale from −3 to 3, where −3 indicated extreme reduction and 3 indicated extreme oxidation. The sensory analysis panel of Burgenland red wine consisted of 7 members, all male, and among the participants, 5 were winemakers with experience in wine tasting and wine closure assessments, while 2 were members of the commercial team with expertise in sensory wines closure evaluations. For each closure type used, panelists ranked the wines on a scale from 1 to 4, where 1 indicated the wine with the most reduction and 4 indicated the wine with the most oxidation.

All panel members underwent continuous training to ensure consistent and reliable results. The training focused on evaluating attributes such as oxidation and reduction, which are key for panel alignment. Panel performance was monitored using the PanelCheck^®^ software (V1.4.2, NOFIMA, Tromsø, Norway), which performs statistical analyses such as Tucker plots and ANOVA, ensuring the accuracy and reliability of the results. The sensory analysis was conducted in tasting sessions using ‘XL5-type’ wine glasses (ISO 3591–1977) [25], with 30 mL wine and covered with watch glass lids. For the Douro red wines, both orthonasal (“aroma”) and retronasal (“palate”) assessments were performed in a single session per closure type. For the Burgenland red wines, orthonasal and retronasal assessments were conducted over two sessions, with sample presentation randomized using a Latin square design.

### 2.6. Determination of the Phenolic Profile of Wines

Following an adaptation of the methodology described by Gao et al. [11], wines were filtered using PTFE filters with a pore diameter of 0.2 µm for UHPLC-UV-MS^n^ analysis. Aliquots of 15 µL were injected into the UHPLC system, which consisted of an Accela 600 LC pump, an Accela autosampler (set to 16 °C), and an Accela 80 Hz photodiode array detector (DAD) (Thermo Fisher Scientific, San Jose, CA, USA). The chromatographic separation was performed on a Hypersil Gold RP C18 column (100 × 2.1 mm, 1.9 µm particle size) preceded by a C_18_ pre-column (2.1 mm I.D.), both from Thermo Fisher Scientific, and maintained at 40 °C. The mobile phase was composed of (A) water (99:1, *v*/*v*) and (B) acetonitrile, both containing 0.1% (*v*/*v*) formic acid. A gradient elution was used at a flow rate of 0.30 mL min^−1^ for 22 min as follows: 1% B from 1 to 2 min; 1–8% B from 2 to 4.5 min; 8–22% B from 4.5 to 14 min; 22–42% B from 14 to 18 min; 42–100% B from 18 to 22 min; 100–1% B from 22 to 26 min and kept at 100% B from 26 to 30 min. Chromatograms were recorded at 280, 320, 360, and 520 nm, and molecular absorption spectra were collected between 210 and 600 nm. The UHPLC system was coupled to an LCQ Fleet ion trap mass spectrometer (Thermo Finnigan, San Jose, CA, USA) equipped with an electrospray ionization (ESI) source. ESI-MS was operated at a spray voltage of 5 kV with a capillary temperature of 315 °C. Nitrogen was used as both sheath and auxiliary gas, with flow rates of 40 and 5 arbitrary units, respectively. The capillary voltages were −35 V (negative mode) and 47 V (positive mode), with tube lens voltages set at −125 V and 115 V, respectively. Collision-induced dissociation (CID) MS^n^ experiments were conducted on mass-selected precursor ions in the *m*/*z* range of 100–2000, with an isolation width of 1.0 mass units. The scan time was set to 100 ms, and the collision energy was 35 arbitrary units, with helium used as the collision gas. Data acquisition was managed through the Xcalibur^®^ data system (Thermo Finnigan, San Jose, CA, USA). Phenolic compound quantification was performed based on peak area at the maximum wavelength absorption, using selected chemical standards to create calibration curves (Table 2). The results for each phenolic compound were expressed in equivalents of the corresponding standard. Concentrations were calculated from triplicate injections (n = 3) per bottle, in a total of 4 bottles per type of closure (4 bottles × 3 replicates, n = 12).

### 2.7. Determination of the Volatile Profile of Wines

The volatile composition of the wines was analyzed using headspace solid-phase microextraction (HS-SPME) coupled with comprehensive two-dimensional gas chromatography and mass spectrometry with a time-of-flight analyzer (GC × GC-ToFMS). In brief, 4 mL of wine, 50 μL of the internal standard 3-octanol (810.81 μg/L), and 1.2 g of sodium chloride were placed in a 12 mL vial and equilibrated at 40.0 ± 0.1 °C for 5 min. The extraction of volatile compounds was performed using a 50/30 µm fused silica fiber coated with divinylbenzene/carboxen/polydimethylsiloxane (DVB/CAR/PDMS). The fiber was inserted into the headspace for 20 min while continuously stirring the sample at 250 rpm. After extraction, the SPME fiber was manually introduced into the GC × GC-ToFMS injector, at 250 °C. A 0.75 mm I.D. glass liner was used, and splitless injection mode was performed (30 s). The system used was a LECO Pegasus 4D GC × GC-ToFMS (LECO, St. Joseph, MI, USA), consisting of an Agilent GC 7890A (Agilent Technologies, Inc., Wilmington, DE, USA) equipped with a dual-stage jet cryogenic modulator (licensed by Zoex) and a secondary oven. A DB-FFAP column (30 m × 0.25 mm I.D., 0.25 μm film thickness, J&W Scientific Inc., Folsom, CA, USA) was used in the first dimension (^1^D), and an Equity-5 column (0.79 m × 0.25 mm I.D., 0.25 μm film thickness, Supelco, Inc., Bellefonte, PA, USA) in the second dimension (^2^D). The carrier gas was helium, flowing at a constant rate of 2.50 mL min^−1^. The oven temperature program began at 40 °C for 3 min, increased to 150 °C at 6 °C min^−1^, and then increased to 230 °C for 2 min at 20 °C min^−1^. The secondary oven was set at 5 °C higher than the primary oven. The MS transfer line and source temperatures were both maintained at 250 °C. The modulation period was 3 s, with a modulator offset 15 °C above the primary oven and modulation pulses alternating between 0.80 s (hot) and 1.70 s (cold). The time-of-flight (ToF) analyzer was operated with a spectrum storage rate of 100 spectra per second. The mass spectrometer was set to electron ionization mode (70 eV), scanning an *m*/*z* range of 30–300.

Automated data processing software ChromaTOF^®^ (V4.72.0.0, LECO) was used to process total ion chromatograms, at a signal-to-noise threshold of 100. The data obtained were transferred into Guineu software (version 0.9, the software source code is published under GNU General Public License that can be downloaded from the internet https://code.google.com/p/guineu/ (accessed on 14 October 2024). This software performs score alignment based on first-dimension retention time (^1^*t*_R_), on second-dimension retention time (^2^*t*_R_), linear retention index (RI) value, spectra, and compound name. For identification purposes, the mass spectrum and retention times of the analytes were compared with existing standards, when available. The mass spectrum of each peak was compared to those existing in mass spectral libraries, including an in-house library of standards and two commercial databases (Wiley 275 and US National Institute of Science and Technology (NIST) V.2.0–Mainlib and Replib). Moreover, a manual analysis of mass spectra was performed using additional information such as RI value, experimentally obtained through the van Den Dool and Kratz RI equation [26]. To determine the RI, a C_8_-C_20_ n-alkanes series was used (solvent n-hexane was used as the C_6_ standard), comparing these values with those reported in the literature for chromatographic columns equivalent to the first-dimension column. Quantification of volatile compounds was achieved using the internal standard method, with concentrations expressed in μg/L 3-octanol equivalents. Three independent aliquots of each wine were analyzed per bottle, in a total of 4 bottles per type of closure (4 bottles × 3 replicates, n = 12).

### 2.8. Statistical Analysis

Univariate analysis was employed to identify potential significant differences between variables across the conditions studied (wines sealed with different closures). This analysis was applied to data sets comprising oxygen transfer rate (OTR), sensory, physicochemical, phenolic, and volatile components. The statistical tests used included nonparametric *t*-tests, multiple *t*-tests, one-way ANOVA (followed by Fisher’s least significant difference tests), and two-way ANOVA (followed by Tukey’s multiple comparison test). These analyses were performed using GraphPad Prism 8.0.1, with a significance threshold of *p* < 0.05. Additionally, clustering analysis were conducted on the volatile compounds and combining all the domains of data (sensory analysis, physicochemical parameters, phenolic compounds, and volatile organic compounds) using MetaboAnalyst 5.0 (web-based software from The Metabolomics Innovation Centre (TMIC), Edmonton, AB, Canada). Prior to the multivariate analyses, the data set was normalized by autoscaling. The results of Douro and Burgenland wines were handled separately.

## 3. Results and Discussion

### 3.1. Determination of the Oxygen Transfer Rate

**Figure 1:**
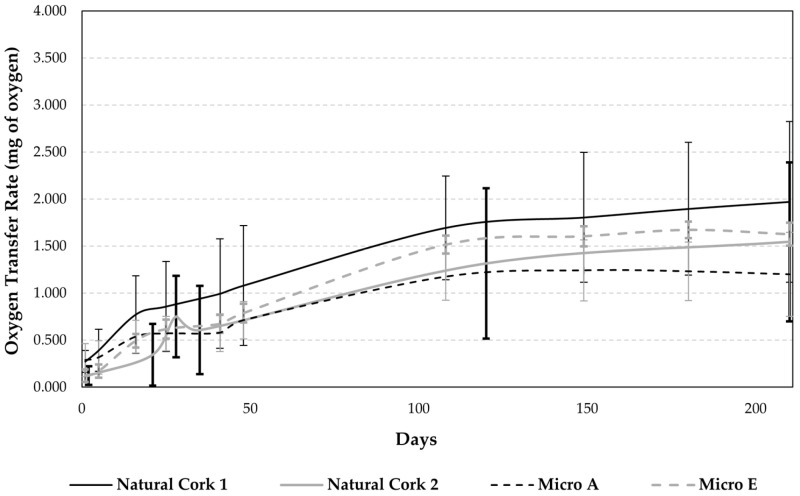
Oxygen transfer rate (OTR), expressed in mg of oxygen, for different stoppers (Natural Cork 1, Natural Cork 2, Micro A, and Micro E), determined over 210 days, under controlled environmental conditions. Error bars represent the standard deviation of five measurements taken per analysis time and closure type. No statistically significant differences (*p* > 0.05) were observed between the four types of stoppers.

The OTR, expressed as milligrams of oxygen per bottle, was monitored over 210 days (Figure 1) for natural and micro-agglomerated cork stoppers. At the end of this period, Natural Cork 1 reached 1.971 ± 0.854 mg of O_2_, Natural Cork 2 achieved 1.546 ± 0.846 mg of O_2_, Micro A recorded 1.200 ± 0.446 mg of O_2_, and Micro E showed 1.627 ± 0.121 mg of O_2_, corresponding to variability, expressed as standard deviation, of 43%, 55%, 37%, and 5%, respectively. Compared with the existing literature, the observed OTR values are within the generally reported range of 1 to 3 mg of O_2_ per year for natural cork stoppers [18,27,28,29], closer to the lower limit of this range. These results can be explained by the duration of the assay (210 days), as the trend lines presented for natural cork stoppers show that OTR values are still increasing. Other studies report that the OTR can fluctuate over the years of storage, with some findings indicating broader ranges of oxygen transfer that extend beyond the values typically observed during short- to medium-term assessments [1,2,27,30,31]. For micro-agglomerated cork stoppers lower OTR values have been reported, ranging from 0.38 to 2.0 mg of O_2_ per year, depending on the stopper dimension [1,9].

Natural Cork 1 exhibited tendentially higher OTR; however, analysis of variance found no statistically significant differences between the stoppers (*p* > 0.05), suggesting similar OTR behavior across stopper types under the study conditions. The Natural Cork 1 presents a lower length (45 × 24 mm—length × diameter), compared with the others (49 × 24 mm), which may explain the tendentially higher OTR values. In fact, factors such as the cork stopper size and cork–glass interface conditions can influence oxygen ingress [18,32]. Namely, the size of the stopper is relevant, as it determines its available surface (diameter) and filter thickness (length) [1,33].

The greater variability among natural cork stoppers is likely attributed to the natural heterogeneous morphology of natural cork, where differences in pore structure and density influence oxygen permeability [1,4,34]. Micro-agglomerated corks, such as Micro A, and especially Micro E, demonstrated lower variability due to their uniform structure derived from adhesive-based manufacturing processes, which allowed for improved control over the gas transference from and to the wine [1]. Also, OTR variability depends on factors such as cork quality, structural uniformity, and sealing conditions [4,29,34,35].

### 3.2. General Physicochemical Characteristics and Sensorial Properties of Wines Sealed with Different Types of Closures

For this section and the following ones, the discussion of the results begins with the data obtained from the Douro blend wine stored for 35 months using natural and micro-agglomerated cork stoppers. In this case, a simpler model was studied, which involves two very common closures used to seal bottles containing red wines, allowing for a detailed assessment of the effects of medium-term storage. After that, we discuss the results obtained from the Burgenland red wines stored for a shorter period of 5 months. This assay included the addition of the screw cap closure recommended for relatively short storage times. The physicochemical properties of wines, including SO_2_ levels, total acidity, volatile acidity, and pH, are key indicators of wines’ quality and stability over time and are presented in Table 3.

According to Table 3, no significant differences (*p* > 0.05) were obtained for all the parameters under study considering the Douro wines bottled with Natural Cork 1 and Micro A, which were stored for 35 months in a horizontal position. Particular attention was devoted to the free and total SO_2_ levels, as these are the main antioxidants used to preserve the wines, providing protection against oxidation and microbial spoilage. The wine oxidation index is determined based on the total SO_2_ levels measurement, which represents both free and bound forms of SO_2_ that react with several wines’ constituents, such as acetaldehyde, anthocyanins, etc. Considering the Douro wines, the free and total SO_2_ levels tend to be slightly higher in wine bottled with Natural Cork 1; however, the data did not provide statistical confirmation of this trend. For the Burgenland assay, which was stored horizontally for 5 months, no significant differences were observed (*p* > 0.05). The SO_2_ levels tended to be slightly lower in wines bottled with the screw cap; however, there are no statistical data to confirm this trend.

The chromatic parameters, expressed by color intensity, shade, and percentage of yellow, red, and blue, for Douro and Burgenland wines sealed with different closures, are presented in Table 4. As shown in this table, no statistically significant differences (*p* > 0.05) were observed between Douro wines bottled with Natural Cork 1 and Micro A. This is consistent with previous studies, which demonstrated no differences were observed between wines bottled with natural and micro-agglomerated cork stoppers [12]. Besides that, for the Burgenland red wines, only slight differences were observed for the wines bottled with a Screw Cap that showed the lowest value of shade (0.864 ± 0.002) and percentage of yellow (40.745 ± 0.063%) and exhibited the highest value of percentage of red (47.169 ± 0.064%), with statistically significant differences (*p* < 0.05).

The sensory analysis evaluated the impact of closure type on the oxireduction level of wines perceived by trained panels, considering both orthonasal and retronasal perceptions (Table 5). For the Douro wines stored for 35 months, a scale from −3 to 3 was used, where −3 indicated extreme reduction, 0 was neutral, and 3 indicated extreme oxidation. As presented in Table 5, for the orthonasal perception, no statistical differences (*p* < 0.05) were observed between wines bottled with different closures. Otherwise, despite the considerable level of dispersion in the responses obtained by this panel, retronasal perception is still possible to observe statistically significant differences between the 2 wines (*p* < 0.05), where wines bottled with Natural Cork 1 exhibited a slightly higher level of oxidation (0.60 ± 0.50) than those sealed with micro-agglomerated Micro A (−0.40 ± −1.00). 

For the Burgenland wines stored for 5 months, a scale from 1 to 4 was used, where 1 represented the most reductive character and 4 the most oxidized character. For orthonasal perception, no statistical differences were observed (*p* > 0.05) between wines bottled with different closures, as previously described for Douro wines, with longer storage times (35 months). Otherwise, for retronasal perception, no significant differences (*p* > 0.05) were observed, with the exception of wines sealed with Micro E and a screw cap (*p* < 0.05). Despite no statistically significant differences being noticed, it is worth highlighting the fact that wines sealed with a screw cap tended to exhibit a more reductive character at a retronasal level.

While the OTR data showed no significant differences among the natural and micro-agglomerated cork stoppers under study (Figure 1), Douro red wines sealed with natural cork stopper for a medium-length storage time seemed to present stronger oxidation characteristics only for retronasal perception, which may be due to variability in oxygen ingress (Natural Cork 1 exhibited tendentially higher OTR). The absence of statistically significant differences in the orthonasal perception, for both wines, can be explained by the fact that aroma perception primarily involves simple volatile compounds present at lower concentrations, which are less affected by the type of closure [19,20]. In contrast, the differences observed in the retronasal perception stem from flavor perception being influenced by more complex interactions between volatile and non-volatile compounds. These differences are likely amplified by the effects of oxygen ingress over time, as oxidative reactions can alter the composition of phenolics and other wine constituents, influencing retronasal flavor characteristics [36,37]. Moreover, retronasal perception integrates the dynamic interaction of aroma and taste during wine consumption, which may better reflect the subtle differences arising from closure type and storage conditions.

### 3.3. Phenolic Profile of Wines Sealed with Different Types of Closures

The phenolic compounds identified in wines, as well as their retention times, the corresponding [M-H]^−^ and (M^+^) ions (for anthocyanins), and the MS*^n^* product ions relevant for their putative identification are listed in Supplementary Material Tables S1 and S2. Additionally, Table 6 and Table 7 present the phenolic compounds that exhibit significant differences (*p* < 0.05) among Douro red wines sealed with different types of closures, and among Burgenland red wines (at least among two wines), respectively.

From the 25 compounds identified and quantified in the Douro red wines (Supplementary Material Table S1), only 8 compounds exhibited significant differences among the wines sealed with different stoppers (1 phenolic acid, 4 flavonols, and 3 anthocyanins), which were present in slightly lower amounts in the wines bottled with Natural Cork 1. However, the observed magnitude of variation is subtle, with fold changes < 0.25-fold (Table 6). The slight changes in the phenolic profile of the wines sealed with different closures may be the result of a balance between migrations from cork-related closures and/or chemical modifications that may occur under post-bottling conditions [11,21]. For instance, an oxidative environment supported by closures may promote oxidative polymerization reactions, which can cause a rapid decrease in free anthocyanins. Flavanols may also be involved in redox reactions [11]. Concerning the polyphenol migration from cork to wine, a previous study showed that natural cork stoppers and agglomerated cork stoppers showed similar behavior, as no significant differences were observed [21]. There is no evidence of significant changes in the total levels of phenolic compounds among wines sealed with different closures. However, considering that Natural Cork 1 tended to exhibit higher OTR compared to Micro A (Figure 1), it may be inferred that OTR may impact the evolution of phenolic compounds in Douro red wine using different types of stoppers. These results can also be correlated with the sensorial analysis, which showed that the wine sealed with Natural Cork 1 exhibited a slightly higher level of oxidation for retronasal perception. However, no obvious association was observed between the evolution of phenolic profiles and the chromatic properties of wines, which seems to make sense, given that the changes are very subtle.

For the short storage assay with Burgenland red wines, there are no clear evolution profiles depending on the type of closure; however, in the case of wines sealed with Natural Cork 2, a tendency for decreasing phenolic compounds concentrations was also observed. Based on the 28 phenolic compounds determined in the Burgenland red wines (Supplementary Material Table S2), it may be pointed out that wines sealed with Natural Cork 2 contained statistically significantly lower amounts (*p* < 0.05—with a magnitude of variation < 0.25-fold) of quercetin-glucuronide, malvidin 3-*O*-glucoside and malvidin-3-*O*-glucoside-4-vinylphenol compared, in particular, with wine sealed with Micro A (Table 7). Despite no significant differences being observed for OTR among the different types of closures, Natural Cork 2 tends to present OTR values similar to those of Micro E, and higher than those of Micro A (Figure 1). Thus, as observed for the medium-length storage assay involving Douro wine, the evolution of phenolic profiles appears to be influenced by the ingress of oxygen. The investigation should be extended beyond 5 months to assess the major possible influences of the different closures on the evolution of phenolic profiles and to evaluate their impact on the sensorial characteristics of wine.

### 3.4. Volatile Profile of Wines Sealed with Different Types of Closures

**Figure 2:**
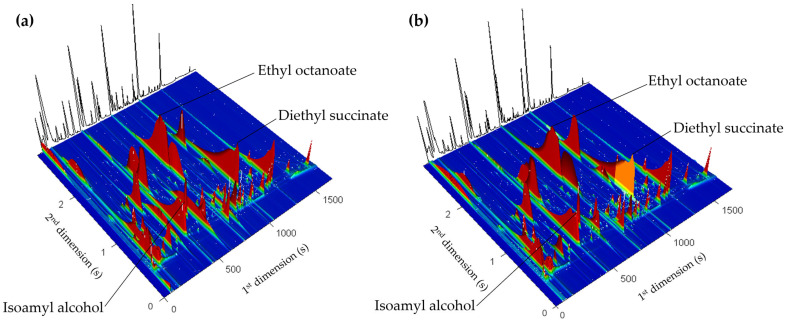
Three-dimensional total ion GC × GC chromatogram of the Douro red wines bottled with (**a**) Natural Cork 1 stopper and (**b**) Micro A. Three major components are marked on the chromatograms: isoamyl alcohol (^1^t_R_ = 435 s and ^2^t_R_ = 0.592 s), ethyl octanoate (^1^t_R_ = 738 s and ^2^t_R_ = 1.864 s) and diethyl succinate (^1^t_R_ = 1032 s and ^2^t_R_ = 0.992 s).

The volatile composition of the wines was assessed using HS-SPME/GC × GC-ToFMS, and 3D total ion current (TIC) chromatograms for Douro wines bottled with Natural Cork 1 and Micro A are provided in Figure 2a,b, respectively, to illustrate the typical chromatograms acquired in this study. This advanced chromatographic technique allows a detailed and sensitive analysis of the volatile compounds, enabling the identification and quantification of complex mixtures with high resolution and enhanced separation efficiency [45]. Both wines’ volatile profiles appear to be dominated by just a few highly concentrated analytes, such as isoamyl alcohol (^1^t_R_ = 435 s and ^2^t_R_ = 0.592 s), ethyl octanoate (^1^t_R_ = 738 s and ^2^t_R_ = 1.864 s) and diethyl succinate (^1^t_R_ = 1032 s and ^2^t_R_ = 0.992 s). In fact, the volatile composition of the two wines appears similar at first glance, as the color scale is biased towards these highly concentrated analytes. However, a detailed examination of the chromatograms was performed to extract data related to the level of chemical complexity within the wines sealed with different closures and to determine analytes that may exhibit noticeable concentration differences between these wines. Data processing and identifying compounds detected by GC × GC-ToFMS is a major challenge, particularly for data processing with a large number of analytes per sample.

In the first step, a data alignment strategy was implemented using a freely available open-source software package, Guineu [46], which was appropriate for handling the large data set acquired by GC × GC-ToFMS. After this step, a data set composed of 548 analytes was obtained for Douro red wines, and 347 analytes were obtained for Burgenland red wines; finally, to reduce the dimension of the data sets and identify analytes that exhibit significant differences between wines bottled with different closures, multiple *t*-tests were performed. A list of compounds usually considered relevant to wine aromas, such as esters, terpenic compounds, and alcohols, was also added to this data set [47]. Also, particular attention was devoted to the analysis of compounds previously associated with oxidative reactions, such as Strecker aldehydes, which were formed through the oxidation of amino acids; norisoprenoids resulting from the oxidative cleavage of carotenoids; and dioxanes [1,13,18,48,49,50]. Furthermore, to better comprehend the interplay between oxidative and reductive reactions in wine, sulfur compounds—key markers of reductive processes—were also investigated [51,52].

**Figure 3:**
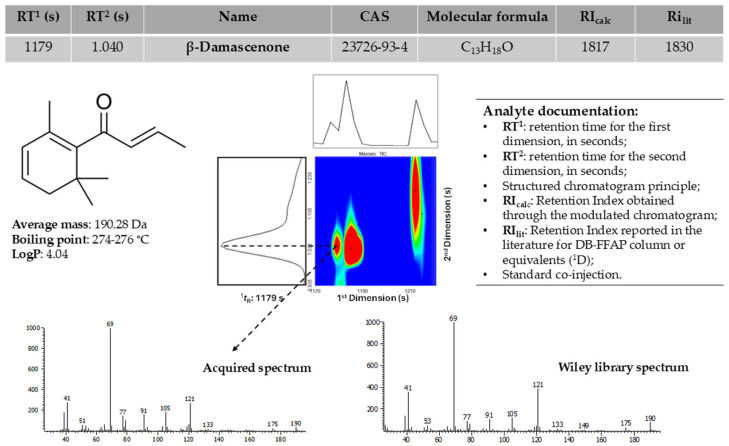
Example of the analyte documentation procedure used to putatively identify the volatile compounds: β-damascenone, presented in wines sealed with different closures, detailing its retention times (on the first and second dimensions), linear retention index, and spectral identification through GC × GC analysis (literature retention index (Ri_lit_) obtained from [53]).

The in-depth analysis of the data involved the identification of analytes present in the datasets established for each assay (Douro and Burgenland red wines), as shown in Figure 3, for β-damascenone, which was identified in wines sealed with different closures. This strategy comprises a comprehensive evaluation of several experimental parameters achieved through GC × GC analysis, as well as data extracted from databases, and the particular physicochemical properties of each molecule. This procedure, although time-consuming, increases confidence in the identification of analytes.

After the previously reported steps, two data sets were established for the two assays: a set of 196 volatile compounds was putatively identified in Douro red wine (Supplementary Material Table S3) and a set of 161 volatile compounds was selected for Burgenland red wine (Supplementary Material Table S4). Given the extent of information obtained regarding the volatile profile of the wines, the results of the Douro and Burgenland red wine assays will be discussed in two separate sub-topics.

#### 3.4.1. Douro Red Wine

The data set of 196 analytes includes compounds from a wide range of chemical families, namely, esters (51), acids (10), alcohols (28), aldehydes (8), aromatic hydrocarbons (10), dioxanes (2), ethers (5), furan derivatives (22), ketones (10), lactones (3), naphthalenes (5), norisoprenoids (3), phenol derivatives (7), sulfur compounds (6), terpenic compounds (17), and others (9). From this data set, 104 (ca. 53%) of the analytes exhibit significant differences among the wines bottled with the two types of closures.

**Figure 4:**
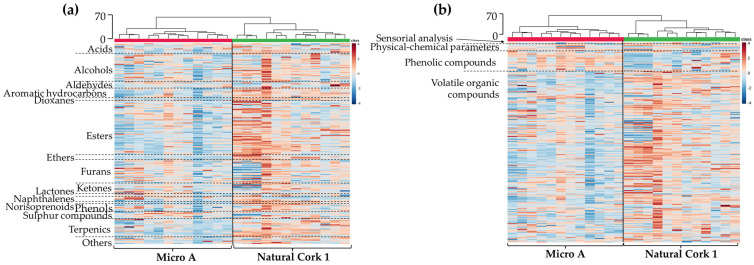
Dendrogram and heatmap representation of Douro red wines sealed with Natural Cork 1 and Micro A stoppers: (**a**) the 196 volatile compounds identified in Supplementary Material Table S3, organized by chemical families separated by dashed lines; and (**b**) the combined information domains, including sensorial analysis, physicochemical parameters, phenolic compounds, and volatile organic compounds. Euclidean distances are included on the dendrogram *Y*-axis. A chromatic scale (from dark blue, minimum, to dark red, maximum) was used for each variable, normalized by autoscaling.

For rapid and visual access to the volatile pattern of the wines sealed with different closures, clustering analysis was performed, and the heatmap and dendrogram show two main clusters corresponding to the wines sealed with Natural Cork 1 and Micro A (Figure 4a). The content of each compound is illustrated through a chromatic scale (from dark blue to dark red, the minimum and the maximum, respectively), and unveiled that wine sealed with Natural Cork 1 exhibited, in general, higher amounts of volatile compounds. However, it is important to point out that, for analytes that present statistically significant differences among wines, on average, the variation magnitude is less than 0.25-fold. Similar clustering was observed (with similar Euclidean distance—70) by using all domains of information (sensory analysis, physicochemical parameters, phenolic compounds, and volatile organic compounds), which also revealed two main clusters corresponding to the wines sealed with Natural Cork 1 and Micro A (Figure 4b). This graphical representation reinforces the information that wines sealed with Natural Cork 1, in general, contain a slightly lower amount of phenolic compounds and a slightly higher quantity of volatile compounds.

A closer look at the volatile composition (Supplementary Material Table S3) highlighted differences between the volatile profiles of wines sealed with different seals, which will be discussed below.

**Figure 5:**
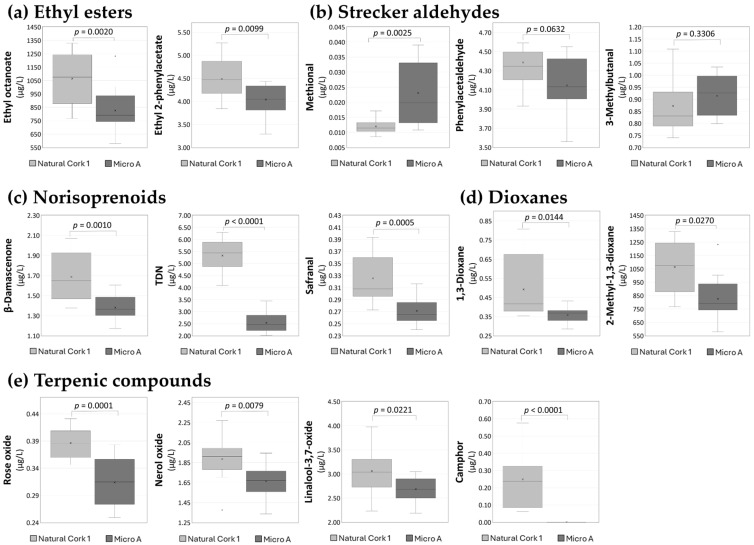
Boxplots of concentrations of (**a**) ethyl esters (ethyl octanoate and ethyl 2-phenylacetate), (**b**) strecker aldehydes (methional, phenylacetaldehyde, and 3-methylbutanal), (**c**) norisoprenoids (β-damascenone, TDN, and safranal), (**d**) dioxanes (1,3-dioxane and 2-methyl-1,3-dioxane), (**e**) terpenic compounds (rose oxide, nerol oxide, linalool-3,7-oxide, and camphor) in Douro red wine bottled with Natural Cork 1 and Micro A closures. The results are expressed in equivalents of 3-octanol (internal standard). We identified statistically significant differences between wines bottled with different stoppers at *p* < 0.05, using multiple *t*-tests conducted using GraphPad prism software. These compounds were selected due to their relevance to wine aroma and their role in oxidative and reductive processes, allowing for a comprehensive evaluation of how closure type influences wine composition.

The esters that significantly contributed to the fruity and floral-like aromas of wines [15] exhibited higher content in the wine sealed with Natural Cork 1, as evidenced by the 31 compounds showing statistically significant differences (*p* < 0.05). Among the ethyl esters (Figure 5a), ethyl 2-phenylacetate also showed slightly higher concentrations in the wine sealed with Natural Cork 1 (4.486 ± 0.426 µg/L) compared to the wine sealed with Micro A (4.039 ± 0.346 µg/L). The preservation of ethyl esters during bottle storage has been a challenge for winemakers, as ethyl esters tend to hydrolyze over time, mostly due to the low pH of wines [14]. As natural cork stoppers were able to better conserve the ethyl ester composition of red wines, it can improve their sensory attributes expected by consumers, as previously reported [13].

Building on the formation of Strecker aldehydes through the oxidation of amino acids, their presence in the volatile profile reflects the ongoing interaction between oxidative and reductive processes in wine. While these compounds are generally considered markers of oxidative conditions, with concentrations typically increasing under oxidative conditions [1], their accumulation patterns are influenced by various factors beyond oxidation alone. For a set of three compounds assigned as Strecker aldehydes (Figure 5b), only methional exhibited slight significant differences between the wines sealed with two closure types, and it was present in higher concentrations in wine sealed with Micro A (0.023 ± 0.011 µg/L) compared with wine sealed with Natural Cork 1 (0.012 ± 0.002 µg/L). Based on its aroma descriptor [54], this result may help explain the aroma of cooked potatoes, which is empirically associated with micro-agglomerated cork stoppers. On the other hand, although it may be indicative of oxidation processes, its content depends on a complex network of factors; namely, its accumulation is modulated by interactions with other components of the wine [55,56].

Three norisoprenoid derivatives were identified in both wines: safranal, β-damascenone, and 1,1,6-trimethyl-1,2-dihydronaphthalene (TDN), which were present in higher concentrations (*p* < 0.05) in the wine sealed with Natural Cork 1 compared to wine bottled with Micro A (Figure 5c). During bottle storage, oxidative degradation of carotenoids can lead to the production of norisoprenoids, and these compounds are released as a result of exposure to oxygen, which facilitates the breakdown of carotenoids, allowing for the formation of these key aroma markers [1,57]. They may contribute to notes associated with saffron-like aromas (safranal) [58], petroleum and kerosene-like aromas (TDN) [22], and floral notes (β-damascenone), enhancing the fruity notes in wine [59].

We also noted the presence of a higher amount (*p* < 0.05) of 1,3-dioxane and 2-methyl-1,3-dioxane in wine sealed with Natural Cork 1 (Figure 5d). Although these compounds are primarily formed during the fermentation process, their concentrations can increase significantly during aging, especially under oxidative conditions [39,60]. The higher levels observed in the wine sealed with Natural Cork 1 may suggest that the stopper material could influence the oxidative environment within the bottle, promoting the formation or release of these compounds over time [1,4]. Dioxanes are usually reported as oxidation markers in aged fortified and white wines, with an odor described as sweet and similar to old port [60].

Among the terpenic compounds identified, 13 exhibited significant differences (*p* < 0.05), with higher concentrations observed in the wine sealed with Natural Cork 1. Among the studied compounds, six of them—limonene, camphor, α-muurolene, cis-calamenene, α-calacorene, and nerolidol—showed fold changes greater than 0.25. Terpenic compounds are crucial to the aroma of wine, contributing to floral, fruity, citrus, and herbal notes [1,61]. Natural corks can release small amounts of these compounds into the wine through migration [4]. This was particularly evident with camphor, a bicyclic monoterpene ketone with a distinctive fresh, minty, and eucalyptus-like aroma [12,13,62]. Interestingly, camphor was exclusively detected in wines sealed with Natural Cork 1 (Figure 5e), aligning with previous findings that identify it as a key marker of natural cork stoppers [4,12,13,63]. Its presence highlights the migration of volatile compounds from cork during bottle storage.

Additionally, three monoterpenol oxides—rose oxide, nerol oxide, and linalool-3,7-oxide—were identified. These compounds are oxidation products of terpenic compounds in wine, making them relevant indicators of oxidative processes [64,65,66]. Rose oxide is characterized by sweet and fruity notes [67], nerol oxide is characterized by greenish-floral aromas [65,68], and linalool-3,7-oxide is characterized by floral notes [67]. Slightly higher levels of these compounds were observed in the wine bottled with Natural Cork 1 compared to those bottled with Micro A, as shown in Figure 5e. As natural cork stoppers were able to increase the amount of the terpenic compounds in wines, which may be released from cork or modified under more oxidative conditions, it can improve their sensory attributes, which will be appreciated by consumers.

#### 3.4.2. Burgenland Red Wine

For the short storage assay with Burgenland red wines, a set of 161 volatile compounds was putatively identified, comprising a wide range of chemical families, including acids (10), alcohols (28), aldehydes (10), aromatic hydrocarbons (3), esters (44), ethers (3), furan derivatives (13), ketones (9), lactones (3), naphthalene compounds (4), norisoprenoids (2), phenol derivatives (7), sulfur compounds (5), terpenic compounds (13), and others (7). Within this data set, 119 (ca. 74%) exhibited significant differences between at least two wines bottled with different closures.

**Figure 6:**
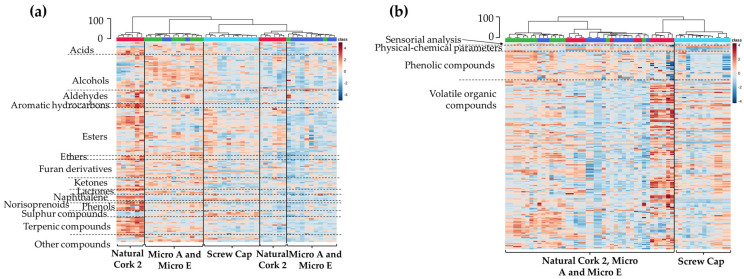
Dendrogram and heatmap representation of Burgenland red wines sealed with Natural Cork 2, Micro A, Micro E, and screw cap closures: (**a**) the 161 volatile compounds identified in Supplementary Material Table S4, organized by chemical families separated by dashed lines; and (**b**) the combined domains of information, including sensorial analysis, physicochemical parameters, phenolic compounds, and volatile organic compounds. Euclidean distances are included on the dendrogram *Y*-axis. A chromatic scale (from dark blue, minimum, to dark red, maximum) was used for each variable, normalized by autoscaling.

A clustering analysis of the volatile patterns in Burgenland wines bottled with different closures was performed, with the heatmap and dendrogram representation revealing five main clusters (Figure 6a), showing that wines sealed with a screw cap seem to be more distinctive than others. Mixing was observed in the branches associated with wines sealed with micro-agglomerated cork stoppers from two producers. For analytes that showed statistically significant differences between at least two wines bottled with different closures, the average magnitude of variation was less than 0.25-fold. In this case, for a short storage period, clustering (with a similar Euclidean distance of 100) using all domains of information (sensory analysis, physicochemical parameters, phenolic compounds, and volatile organic compounds) revealed two main clusters: one comprising wines sealed with Natural Cork 2, Micro A, and Micro E, and the other comprising wine sealed with a screw cap (Figure 6b). This graphical representation highlights that the volatile composition is particularly sensitive to changes induced by closures, because when more domains of information are combined, there is less distinction between wines sealed with different closures (Figure 6b).

**Figure 7:**
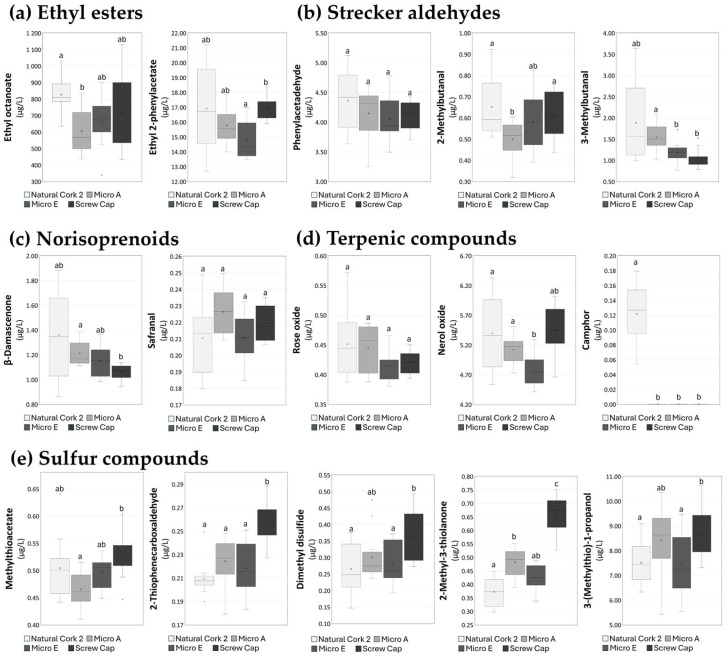
Boxplots of concentrations of (**a**) ethyl esters (ethyl octanoate and ethyl 2-phenylacetate), (**b**) Strecker aldehydes (phenylacetaldehyde, 2-methylbutanal, and 3-methylbutanal), (**c**) norisoprenoids (safranal and β-damascenone), (**d**) terpenic compounds (rose oxide, nerol oxide, and camphor), (**e**) sulfur compounds (methylthioacetate, 2-thiophenecarboxaldehyde, dimethyl disulfide, 2-mehtyl-3-thiolanone, and 3-(methylthio)-1-propanol) determined in Burgenland red wine bottled with Natural Cork 2, Micro A, Micro E and screw cap closures. The results expressed in equivalents of 3-octanol (internal standard). Different superscript lowercase letters in a row represent statistically significant differences between wines bottled with different closures at *p* < 0.05, using multiple *t*-tests and GraphPad prism. These compounds were selected due to their relevance to wine’s aroma and their role in oxidative and reductive processes, allowing for a comprehensive evaluation of how closure type influences wine composition.

A detailed analysis of the volatile composition (Supplementary Material Table S4) revealed distinct patterns in the ester profile across wines sealed with different closures, once again highlighting the influence of stoppers on the volatile profile. These compounds, which are known to contribute to the floral and fruity notes in wine [15], were more abundant in the wine bottled with Natural Cork 2. This is evident from the slightly higher levels of ethyl octanoate (826.627 ± 121.210 µg/L) and ethyl hexanoate (374.064 ± 81.606 µg/L) in this wine compared to the other wines. Additionally, among the ethyl esters (Figure 7a), ethyl 2-phenylacetate also exhibited a slightly higher concentration in the wine sealed with Natural Cork 2 (16.910 ± 2.894 µg/L). These data confirm that natural cork stoppers may contribute to the preservation of ethyl esters in red wines; the results for Douro wine are also considered.

Attention was also devoted to Strecker aldehydes, which, as referred above, are compounds potentially associated with oxidative phenomena [69], including phenylacetaldehyde, 2-methylbutanal, and 3-methylbutanal (Figure 7b). Due to the high variability observed, namely for wine sealed with Natural Cork 2, the results obtained for this short storage in-bottle do not show noticeable differences associated with the closure type.

Two norisoprenoids, safranal, and β-damascenone, were detected in all the studied Burgenland red wines (Figure 7c). For this short storage time in the bottle, differences among the wines sealed with different closures are not so clear. Statistically significant differences were only observed for β-damascenone, with the wine sealed with Micro A showing a slightly higher concentration (1.213 ± 0.099 µg/L) compared to the wine sealed with screw cap (1.058 ± 0.061 µg/L), despite both compounds being present at low levels overall.

For the terpenic compounds, with the exception of camphor, the difference in the terpenic profile between wines sealed with different closures is not particularly noticeable (Figure 7d). Despite this, the wines sealed with Natural Cork 2 exhibited a high variability among bottles (Figure 6a); in general, they exhibited the highest total content of terpenic compounds (31.251 ± 3.083 µg/L), while the total terpenic content of the wines sealed with the other closures ranged between ca. 20.972 and 27.931 µg/L (Supplementary Material Table S4). As observed from the Douro assay, camphor was identified exclusively in the wine sealed with Natural Cork 2, reinforcing the claim that this compound was released into the wine via natural cork stoppers [12]. Although no significant differences were observed for rose oxide among the terpenic oxide types, nerol oxide demonstrated a slight statistically significant difference between the wine bottled with Natural Cork 2 (5.395 ± 0.637 µg/L) and the wine bottled with Micro E (4.768 ± 0.275 µg/L), showcasing potential variations that may arise as a result of the closure type.

Sulfur compounds represent another group of volatile molecules worth noting, as they are reduction-driven volatiles present in the sample. While some sulfur compounds contribute positively to the wine’s aroma profile, others are associated with reductive aromas, often perceived as sensory defaults [17]. Five sulfur compounds were identified in this study: methylthioacetate, 2-thiophenecarboxaldehyde, dimethyl disulfide, 2-methyl-3-thiolanone, and 3-(methylthio)-1-propanol (also known as methionol), all of which exhibited slightly higher concentrations in wines sealed with a screw cap (Figure 7e). The greater concentrations of these sulfur compounds in wines sealed with screw caps may indicate a more pronounced reductive environment. The increased presence of methionol (8.697 ± 0.957 µg/L), for example, is often associated with off-flavors such as potato, soup, or cooked cabbage [70], which could be perceived as undesirable in wines with excessive reductive aromas.

## 4. Conclusions

This study provides new insights into the impact of the closure on the modulation of the red wines’ characteristics during short- and medium-term bottle storage. In fact, the red wine–closure pairing, which has been less studied compared to pairings involving white and sparkling wines, was shown to create distinct environments within the bottles. Oxidative phenomena were slightly more pronounced in wines sealed with natural cork, as evidenced by both sensory and physicochemical data. Terpenic compounds, norisoprenoids, and esters, which contribute to floral and fruity notes, were more pronounced in wines sealed with natural cork, suggesting that these types of closures better preserve or increase the prevalence of compounds associated with sensory attributes appreciated by wine consumers. Moreover, these closure-driven differences became more evident over time, as demonstrated by oxidative–reductive reactions, phenolic oxidative polymerization, and the migration of compounds from the cork stopper to wines, such as camphor. Red wines sealed with screw caps appeared to exhibit the most notable differences among the set under study, indicating a more reductive environment.

The sensory analysis in this study was focused on evaluating the oxireduction level through both orthonasal and retronasal perceptions in wines, which could represent a limitation. This could be overcome through descriptive sensory analysis, allowing the characterization of floral and fruity aromas, among others, which could then be correlated with volatile compounds. 

In conclusion, pairing wine with a specific type of closure can serve as an oenological tool, and this is supported by physicochemical data. These findings were underpinned by advanced chromatographic equipment and the integration of various information domains, employing statistical tools that lay the foundation for developing predictive models.

## Figures and Tables

**Table 1:** List of natural cork stoppers and alternatives used in this study, their respective designations, dimensions, and additional notes.

Closure Type	Designation	Dimension Length and Diameter (mm)	Additional Notes
Natural cork stopper	Natural Cork 1	45 × 24	Surface treatment with paraffin emulsion silicone elastomer blend
Natural cork stopper	Natural Cork 2	49 × 24	Surface treatment with paraffin emulsion and silicone elastomer blend
Micro-agglomerated cork stopper	Micro A	49 × 24	Cork granules with a polyurethane-based binder and treated with aqueous emulsion of silicones and synthetic waxes. Microspheres include acrylonitrile, methylacrylate, methacrylonitrile, sodium 2-cyanoethanesulfonate, sulfite, sodium 3-methoxy-3-oxopropane-1-sulfonate, and hexadecanol
Micro-agglomerated cork stopper	Micro E	49 × 24	Cork granules treated with silicone elastomer blend
Screw Cap Tin Saran closure	Screw Cap	60 × 30	Tin (metal) layer and poly(vinylidene chloride) (PVDC)

**Table 2:** Calibration data used for the quantification of phenolic compounds in red wines from Douro and Burgenland regions.

Compound	Concentration Range (mg/L)	Calibration Curve ^a^	r^2^	LOD (mg/L)	LOQ (mg/L)
Gallic acid	2.60–83.20	y = 1025.2x − 820.43	0.9998	1.84	5.58
Caffeic acid	0.82–61.20	y = 2697.2x − 6.4998	1.0000	0.32	0.98
Ellagic acid	0.71–35.40	y = 1197.2x − 416.12	0.9999	0.37	1.12
Vanillin	0.88–44.00	y = 2492.8x − 916.61	0.9999	0.82	2.48
Catechin	5.10–66.30	y = 323.99x − 416.38	0.9994	2.47	7.48
Narigenin	1.08–37.80	y = 1484.1x + 734.29	0.9996	1.20	3.64
Quercetin	0.27–32.40	y = 2080.7x + 355.79	1.0000	0.06	0.18
Cyanidin chloride	1.00–80.00	y = 846.03x − 375.37	1.0000	0.51	1.56

^a^ y = peak area, x = concentration in mg/L, r^2^—correlation coefficients, LOD—Limit of Detection, LOQ—Limit of Quantification.

**Table 3:** Physicochemical parameters (total acidity, volatile acidity, volatile acidity deduced from SO_2_, SO_2_ free, SO_2_ total, and pH) of the Douro and Burgenland red wines stored for 35 and 5 months, respectively. The results are expressed as the averages of 4 bottles × 3 replicates (n = 12) ± the standard deviation.

Parameter	Douro Red Wine		Burgenland Red Wine			
	Natural Cork 1	Micro A	Natural Cork 2	Micro A	Micro E	Screw Cap
Total acidity (g/L tartaric acid)	5.60 ± 0.06 ^a^	5.55 ± 0.06 ^a^	4.766 ± 0.06 ^a^	4.779 ± 0.06 ^a^	4.805 ± 0.06 ^a^	4.751 ± 0.06 ^a^
Volatile acidity ded. SO_2_ (g/L acetic acid)	0.76 ± 0.05 ^a^	0.76 ± 0.05 ^a^	0.717 ± 0.05 ^a^	0.711 ± 0.05 ^a^	0.714 ± 0.05 ^a^	0.684 ± 0.05 ^a^
SO_2_ free (mg SO_2_/L)	22 ± 6 ^a^	19 ± 6 ^a^	28 ± 6 ^a^	30 ± 6 ^a^	29 ± 6 ^a^	26 ± 6 ^a^
SO_2_ total (mg SO_2_/L)	56 ± 8 ^a^	53 ± 8 ^a^	83 ± 8 ^a^	91 ± 8 ^a^	85 ± 8 ^a^	77 ± 8 ^a^
pH	3.76 ± 0.04 ^a^	3.79 ± 0.02 ^a^	3.70 ± 0.01 ^a^	3.70 ± 0.02 ^a^	3.70 ± 0.01 ^a^	3.69 ± 0.01 ^a^

Different superscript lowercase letters in a row represent statistically significant differences between bottles sealed with different closures at *p* < 0.05. Statistical significance was assessed using two-way ANOVA followed by Tukey’s comparison test. Analyses were conducted separately for each set of samples, evaluating differences exclusively within the Douro samples and, independently, within the Burgenland samples.

**Table 4:** Color intensity (I), shade (N), and percentage of yellow, red, and blue of the Douro and Burgenland red wines stored for 35 and 5 months, respectively. The results are expressed as the averages of 4 bottles × 3 replicates (n = 12) ± the standard deviation.

Chromatic Parameter	Douro Red Wine		Burgenland Red Wine			
	Natural Cork 1	Micro A	Natural Cork 2	Micro A	Micro E	Screw Cap
I (Abs_420_ + Abs_520_ + Abs_620_)	12.167 ± 0.384 ^a^	12.333 ± 0.181 ^a^	8.730 ± 0.137 ^a^	8.785 ± 0.068 ^a^	8.823 ± 0.091 ^a^	8.794 ± 0.158 ^a^
N (Abs_420_/Abs_520_)	0.848 ± 0.005 ^a^	0.854 ± 0.009 ^a^	0.878 ± 0.002 ^a^	0.873 ± 0.002 ^b^	0.876 ± 0.002 ^a^	0.864 ± 0.002 ^c^
%yellow (Abs_420_/I × 100)	39.097 ± 0.157 ^a^	39.153 ± 0.140 ^a^	41.112 ± 0.095 ^a^	40.975 ± 0.053 ^b^	41.082 ± 0.053 ^a^	40.745 ± 0.063 ^c^
%red (Abs_520_/I × 100)	46.100 ± 0.298 ^a^	45.911 ± 0.387 ^a^	46.830 ± 0.057 ^a^	46.961 ± 0.038 ^b^	46.879 ± 0.033 ^c^	47.169 ± 0.064 ^d^
%blue (Abs_620_/I × 100)	14.803 ± 0.383 ^a^	14.936 ± 0.266 ^a^	12.057 ± 0.102 ^ab^	12.065 ± 0.027 ^ab^	12.039 ± 0.025 ^a^	12.086 ± 0.035 ^b^

Different superscript lowercase letters in a row represent statistically significant differences between bottles sealed with different closures at *p* < 0.05. Statistical significance was assessed using Two-Way ANOVA followed by Tukey’s comparison test. Analyses were conducted separately for each set of samples, evaluating differences exclusively within the Douro samples and, independently, within the Burgenland samples.

**Table 5:** Sensory analysis of the oxireduction level of Douro red wines bottle with Natural Cork 1 and Micro A stored for 35 months and Burgenland red wines with Natural Cork 2, Micro A, Micro E, and Screw Cap stored for 5 months.

Parameter	Douro Red Wines		Burgenland Red Wines			
	Natural Cork 1	Micro A	Natural Cork 2	Micro A	Micro E	Screw Cap
Sensation of oxireduction odor (orthonasal)	0.10 ± 1.33 ^a^	−0.10 ± 0.94 ^a^	2.0 ± 1.1 ^a^	2.8 ± 2.1 ^a^	3.2 ± 0.9 ^a^	2.0 ± 0.8 ^a^
Sensation of oxireduction flavor (retronasal)	0.60 ± 0.50 ^a^	−0.40 ± −1.00 ^b^	2.4 ± 1.2 ^ab^	2.4 ± 1.1 ^ab^	3.4 ± 0.6 ^a^	1.7 ± 0.7 ^b^

Different superscript lowercase letters in a row represent statistically significant differences between bottles sealed with different closures at *p* < 0.05, as determined using Friedman’s test and Mann–Whitney: Douro wines (n = 10 tasters, averaged ± standard deviation) and Burgenland wines (n = 14 tasters, made twice, averaged ± standard deviation). It is important to note that the two panels used different sensory scales: the Douro panel used a scale from −3 to 3 for sensory perception, while the Burgenland panel used a scale from 1 to 4.

**Table 6:** Concentration (mg/L) of phenolic compounds exhibiting significant differences (*p* < 0.05) in Douro red wines sealed with Natural Cork 1 and Micro A, determined by UHPLC-DAD-MS. The table includes the retention time (*t*_R_), molecular ion (*m*/*z*), and respective fragmentation data. For anthocyanins, the molecular ion is expressed in positive mode.

*t_R_* (min)	Phenolic Compound	[M-H]^−^ (*m*/*z*)	MS^2^ Product Ions (*m*/*z*)	Natural Cork 1	Micro A
	***Phenolic acids***				
12.68	Ellagic acid ^a^	301	257 (100), 229 (70) [38]	3.49 ± 0.44 ^a^	4.36 ± 0.31 ^b^
	***Flavonols***				
13.56	Quercetin-glucuronide ^b^	477	301 (100), 283 (10) [39,40]	3.67 ± 0.36 ^a^	4.12 ± 0.20 ^b^
13.90	Laricitrin-3-glucoside ^b^	493	331 (100) [41]	1.16 ± 0.16 ^a^	1.48 ± 0.13 ^b^
16.11	Quercetin-glucoronide-glucoside ^b^	639	477 (100) [39]	1.16 ± 0.14 ^a^	1.44 ± 0.19 ^b^
16.51	Dihydrokaempferol-3-rhamnoside ^b^	433	287 (100) [41]	0.45 ± 0.14 ^a^	0.60 ± 0.11 ^b^
	***Anthocyanins***	**(M^+^)**			
10.91	Malvidin-3-*O*-glucoside ^c^	493	331 (100) [42]	6.31 ± 1.04 ^a^	7.19 ± 0.34 ^b^
13.42	Petunidin-3-*O*-glucoside ^c^	479	317 (100) [42]	3.77 ± 0.55 ^a^	4.17 ± 0.31 ^b^
18.25	Peonidin-3-O-(6-*O*-p-coumaroyl)-glucoside ^c^	609	301 (100) [42]	3.00 ± 0.49 ^a^	2.32 ± 0.35 ^b^

***t*_R_**—retention time, **[M-H]^−^**—molecular ion at negative mode, **(M^+^)**—molecular ion at positive mode. Different superscript lowercase letters in a row represent statistically significant differences between wines bottled with different closures at *p* < 0.05, using multiple *t*-tests and GraphPad prism software. The results are expressed as the averages of 4 bottles × 3 replicates (n = 12) ± the standard deviation. **^a^** Expressed in equivalents of ellagic acid. **^b^** Expressed in equivalents of quercetin. **^c^** Expressed in equivalents of cyanidin chloride.

**Table 7:** Concentration (mg/L) of phenolic compounds exhibiting significant differences (*p* < 0.05) at least between two Burgenland red wines sealed with Natural Cork 2, Micro A, Micro E, and screw cap, determined by UHPLC-DAD-MS. The table includes the retention time (*t*_R_), molecular ion (*m*/*z*), and respective fragmentation data. For anthocyanins, the molecular ion is expressed in positive mode.

*t*_R_ (min)	Phenolic Compound	[M-H]^−^ (*m*/*z*)	MS^2^ product ions (*m*/*z*)	Natural Cork 2	Micro A	Micro E	Screw Cap
	***Phenolic acids***						
7.03	Coutaric acid ^a^	295	163 (100) [39]	17.64 ± 2.14 ^ab^	18.35 ± 0.32 ^a^	14.31 ± 2.84 ^b^	17.91 ± 0.56 ^ab^
12.68	Ellagic acid ^b^	301	257 (100); 229 (70) [38]	8.19 ± 2.50 ^ab^	10.19 ± 0.62 ^ab^	10.49 ± 0.70 ^a^	9.35 ± 0.78 ^b^
	***Flavonols***						
13.36	Quercetin-glucuronide ^c^	477	301 (100), 283 (10) [39,40]	17.78 ± 1.49 ^a^	19.24 ± 0.77 ^b^	19.07 ± 0.62 ^ab^	18.12 ± 1.43 ^ab^
15.39	Isorhamnetin-hexoside ^c^	477	315 (100) [43]	7.24 ± 4.92 ^ab^	10.57 ± 0.41 ^ab^	10.31 ± 0.43 ^a^	11.41 ± 0.93 ^b^
	***Anthocyanins***	**(M^+^)**					
11.04	Malvidin 3-*O*-glucoside ^d^	493	331 (100) [42]	1.84 ± 0.18 ^a^	2.34 ± 0.27 ^b^	2.33 ± 0.49 ^ab^	1.82 ± 0.11 ^a^
17.80	Peonidin-3,5-*O*-diglucoside ^d^	625	463 (100), 301 (20) [42]	2.30 ± 0.31 ^a^	2.59 ± 0.34 ^ab^	2.26 ± 0.17 ^a^	2.93 ± 0.38 ^b^
18.44	Malvidin-3-*O*-glucoside-4-vinylphenol ^d^	609	447 (100) [44]	4.37 ± 0.62 ^a^	5.02 ± 0.67 ^b^	3.94 ± 0.57 ^a^	4.50 ± 0.47 ^ab^

***t*_R_**—retention time, **[M-H]^−^**—molecular ion in negative mode, **(M^+^)**—molecular ion in positive mode. Different superscript lowercase letters in a row represent statistically significant differences between wines bottled with different closures at *p* < 0.05, using multiple *t*-tests and GraphPad prism. The results are expressed as the averages of 4 bottles × 3 replicates (n = 12) ± the standard deviation. **^a^** Expressed in equivalents of caffeic acid. **^b^** Expressed in equivalents of ellagic acid. **^c^** Expressed in equivalents of quercetin. **^d^** Expressed in equivalents of cyanidin chloride.

## Data Availability

The original contributions presented in this study are included in the article/Supplementary Material. Further inquiries can be directed to the corresponding author.

## Supplementary Materials

The following supporting information can be downloaded at https://www.mdpi.com/article/10.3390/foods14050783/s1, Table S1. Concentration (mg/L) of phenolic compounds determined in the Douro red wine (bottled with Natural Cork 1 and Micro A closures), by UHPLC-DAD-MS, including retention time (*t*_R_), molecular ion (*m*/*z*), and respective MS*^n^* product ions relevant for their putative identification. For the anthocyanins, the molecular ion is expressed in the positive mode. The compounds marked in bold present statistically significant differences between the wines (*p* < 0.05); Table S2. Concentration (mg/L) of phenolic compounds detected in the Burgenland red wine (bottled with Natural Cork 2, Micro A, Micro E, and screw cap closures), by UHPLC-DAD-MS, including retention time (*t*_R_), molecular ion (*m*/*z*), and respective MS*^n^* product ions relevant for their putative identification. For the anthocyanins, the molecular ion is expressed in the positive mode. The compounds marked in bold present statistically significant differences at least between two wines (*p* < 0.05); Table S3. List of volatile compounds detected in the Douro red wine sealed with Natural Cork 1 and Micro A closures (stored for 35 months until analysis, in a horizontal position), using HS-SPME/GC × GC-ToFMS, including relevant chromatographic data used to assess compounds identification. The compounds marked in bold present statistically significant differences between the wines (*p* < 0.05); Table S4. List of volatile compounds detected in the Austria red wine sealed with Natural Cork 2, Micro A, Micro E and screw cap stoppers (stored for 5 months until analysis, in a horizontal position), using HS-SPME/GC × GC-ToFMS, including relevant chromatographic data used to assess compounds identification. The compounds marked in bold present statistically significant differences at least between two wines (*p* < 0.05). Refs. [26,38,39,40,41,42,43,44,53,71,72,73,74,75,76,77,78,79,80,81,82,83,84,85,86,87,88,89,90,91,92,93,94,95,96,97,98,99,100,101,102,103,104,105,106,107,108,109,110,111,112,113,114,115,116,117,118,119,120,121,122,123,124,125,126,127,128,129,130,131,132,133,134,135,136,137,138,139,140,141,142,143,144,145,146,147,148,149,150,151,152,153,154,155,156,157,158,159,160] are cited in Supplementary Materials.
