# Supplementary material for: Pairing Red Wine and Closure: New Achievements from Short-to-Medium Storage Time Assays
Source: Foods. 2025 Feb 25;14(5):783. doi: 10.3390/foods14050783 (PMC11899619; doi:10.3390/foods14050783)
Supplement: Supplementary file 1 [file foods-14-00783-s001.zip › foods-3482334-supplementary.pdf]

Supplementary Material

Table S1. Concentration (mg/L) of phenolic compounds determined in the Douro red wine (bottled with Natural Cork 1 and Micro A closures), by UHPLC-DAD-MS, including retention time (t_R), molecular ion (*m*/*z*), and respective MS^n product ions relevant for their putative identification. For the anthocyanins, the molecular ion is expressed in the positive mode. The compounds marked in bold present statistically significant differences between the wines ($p < 0.05$).

t_R (min)	Coumpound name	[M-H] (*m*/*z*)	MS^2 product ions (*m*/*z*)	Natural Cork1	Micro A
	Phenolic acids				
1.95	Gallic acid[a]	169	125 (100) [38]	34.46 ± 4.39[a]	35.55 ± 3.88[a]
3.52	Photocatechuic acid[a]	153	109 (100) [71]	18.40 ± 2.52[a]	18.01 ± 1.66[a]
7.03	Coutaric acid[b]	295	163 (100) [39]	3.32 ± 0.33[a]	3.72 ± 0.92[a]
8.52	Caffeic acid[b]	179	135 (100) [71]	21.32 ± 2.71[a]	21.81 ± 1.58[a]
10.69	*p*-Coumaric acid[b]	163	119 (100) [71]	24.65 ± 2.77[a]	24.53 ± 1.66[a]
12.68	**Ellagic acid**[c]	301	257 (100), 229 (70) [38]	3.49 ± 0.44[a]	4.36 ± 0.31[b]
	Aldehydes				
9.73	Vanillin[d]	151	136 (100) [39]	7.35 ± 2.21[a]	8.29 ± 0.88[a]
	Flavan-3-ols				
8.00	Catechin[e]	289	245 (100), 205 (40), 179 (20) [39,40]	29.75 ± 4.46[a]	29.77 ± 3.03[a]
9.66	Procyanidin B1[e]	577	451 (100), 425 (70), 407 (20) [40,72]	44.61 ± 8.25[a]	48.85 ± 5.87[a]
	Flavanone				
16.44	Narigenin hexose derivative[f]	597	435 (100) [39]	5.77 ± 1.14[a]	5.28 ± 0.87[a]
	Flavonols				
13.56	**Quercetin-glucuronide**[g]	477	301 (100), 283 (10) [39,40]	3.67 ± 0.36[a]	4.12 ± 0.20[b]
13.90	**Laricitrin-3-glucoside**[g]	493	331 (100) [41]	1.16 ± 0.16[a]	1.48 ± 0.13[b]
15.39	Myricetin[g]	317	179 (100), 151 (40) [40,73]	7.26 ± 0.88[a]	7.60 ± 0.28[a]
15.68	Syringetin-3-glucoside[g]	507	345 (100) [74]	0.49 ± 0.12[a]	0.57 ± 0.07[a]
16.11	**Quercetin-glucoronide-glucoside**[g]	639	477 (100) [39]	1.16 ± 0.14[a]	1.44 ± 0.19[b]
16.51	**Dihydrokaempferol-3-rhamnoside**[g]	433	287 (100) [41]	0.45 ± 0.14[a]	0.60 ± 0.11[b]

	Anthocyanins	**(M^+)**			
9.62	Peonidin-3-*O*-acetylglucoside[h]	505	301 (100) [75]	2.04 ± 0.35^{a}	2.15 ± 0.18^{a}
10.71	Cyanidin-3-*O*-p-glucoside[h]	449	287 (100) [42]	5.74 ± 0.61^{a}	6.07 ± 0.66^{a}
10.91	**Malvidin-3-*O*-glucoside[h]**	493	331 (100) [42]	6.31 ± 1.04^{a}	7.19 ± 0.34^{b}
13.42	**Petunidin-3-*O*-glucoside[h]**	479	317 (100) [42]	3.77 ± 0.55^{a}	4.17 ± 0.31^{b}
14.19	Malvidin-3-*O*-acetylglucoside[h]	535	331 (100) [42]	4.25 ± 0.79^{a}	4.47 ± 0.33^{a}
16.01	Cyadinin-3-*O*-p-coumarylglucoside[h]	595	415 (20), 287 (100) [75,76]	4.67 ± 0.52^{a}	4.67 ± 0.41^{a}
17.05	Malvidin-3-glucoside-p-coumarate[h]	639	331 (100) [42]	2.38 ± 0.46^{a}	2.41 ± 0.46^{a}
17.43	Peonidin-3,5-*O*-diglucoside[h]	625	463 (100), 301 (20) [42]	3.06 ± 0.67^{a}	3.31 ± 0.64^{a}
18.25	**Peonidin-3-*O*-(6-*O*-p-coumaroyl)-glucoside[h]**	609	301 (100) [42]	3.00 ± 0.49^{a}	2.32 ± 0.35^{b}

t_R – retention time, **$[M-H]^-$** - molecular ion at negative mode, **(M^+)** – molecular ion at positive mode. Different superscript lowercase letters in a row represent statistically significant differences between wines bottled with different stoppers ($p < 0.05$), using Multiple t-tests in GraphPad prism. The results are expressed as the averages of 4 bottles × 3 replicates (n=12) ± the standard deviation. [a] Expressed in equivalents of gallic acid. [b] Expressed in equivalents of caffeic acid. [c] Expressed in equivalents of ellagic acid. [d] Expressed in equivalents of vanillin. [e] Expressed in equivalents of catechin. [f] Expressed in equivalents of narigenin. [g] Expressed in equivalents of quercetin. [h] Expressed in equivalents of cyanidin chloride.

Table S2. Concentration (mg/L) of phenolic compounds detected in the Burgenland red wine (bottled with Natural Cork 2, Micro A, Micro E, and Screw Cap closures), by UHPLC-DAD-MS, including retention time (t_R), molecular ion (*m/z*), and respective MS^n product ions relevant for their putative identification. For the anthocyanins, the molecular ion is expressed in the positive mode. The compounds marked in bold present statistically significant differences at least between two wines ($p < 0.05$).

t_R (min)	Phenolic compound	$[M-H]^-$ (*m/z*)	MS^2 product ions (*m/z*)	Natural Cork 2	Micro A	Micro E	Screw Cap
	Phenolic acids						
1.84	Gallic acid[a]	169	125 (100) [38]	63.59 ± 5.80[a]	66.53 ± 2.59[a]	66.94 ± 3.29[a]	66.25 ± 4.84[a]
4.69	Caftaric acid[b]	311	179 (100) [39]	46.91 ± 5.83[a]	49.85 ± 1.39[a]	48.94 ± 4.58[a]	47.82 ± 1.46[a]
7.03	**Coutaric acid[b]**	295	163 (100) [39]	17.64 ± 2.14[ab]	18.35 ± 0.32[a]	14.31 ± 2.84[b]	17.91 ± 0.56[ab]
8.52	Caffeic acid[b]	179	135 (100) [71]	20.21 ± 2.59[a]	21.91 ± 0.46[a]	21.13 ± 3.04[a]	21.59 ± 1.94[a]
10.69	*p*-coumaric acid[b]	163	119 (100) [71]	14.53 ± 1.60[a]	15.61 ± 0.31[a]	14.80 ± 1.58[a]	15.32 ± 1.29[a]
12.68	**Ellagic acid[c]**	301	257 (100); 229 (70) [38]	8.19 ± 2.50[ab]	10.19 ± 0.62[ab]	10.49 ± 0.70[a]	9.35 ± 0.78[b]
	Flavan-3-ols						
7.95	Catechin[d]	289	245 (100), 205 (40), 179 (20) [39,40]	15.33 ± 2.20[a]	14.87 ± 3.26[a]	13.36 ± 2.17[a]	14.65 ± 3.13[a]
9.14	Procyanidin B1[d]	577	451 (100), 425 (70) [40,72]	56.09 ± 2.44[a]	55.94 ± 4.14[a]	56.14 ± 3.15[a]	55.00 ± 5.11[a]
9.59	Epicatechin[d]	289	245 (100), 205 (40), 179 (20) [39,40]	56.39 ± 7.91[a]	53.31 ± 7.34[a]	52.20 ± 7.55[a]	55.09 ± 4.18[a]
	Flavonols						
9.73	Kaempferol-glucoside[e]	447	285 (100) [39]	1.31 ± 0.25[a]	1.21 ± 0.20[a]	1.27 ± 0.29[a]	1.10 ± 0.12[a]
11.91	Myricetin-3-*O*-galactoside[e]	479	316 (100), 317 (40), 271 (5) [40]	10.73 ± 0.91[a]	10.84 ± 0.25[a]	10.95 ± 0.39[a]	10.46 ± 0.57[a]
13.36	**Quercetin-glucuronide[e]**	477	301 (100), 283 (10) [39,40]	17.78 ± 1.49[a]	19.24 ± 0.77[b]	19.07 ± 0.62[ab]	18.12 ± 1.43[ab]
13.81	Laricitrin-3-glucoside[e]	493	331 (100) [41]	6.08 ± 1.57[ab]	6.45 ± 0.14[a]	6.39 ± 0.57[ab]	5.70 ± 0.49[b]
15.09	Myricetin[e]	317	179 (100), 151 (40) [40,73]	0.95 ± 0.18[a]	1.08 ± 0.11[a]	1.03 ± 0.17[a]	0.90 ± 0.15[a]
15.39	**Isorhamnetin-hexoside[e]**	477	315 (100) [43]	7.24 ± 4.92[ab]	10.57 ± 0.41[ab]	10.31 ± 0.43[a]	11.41 ± 0.93[b]
15.53	Syringetin-3-glucoside[e]	507	345 (100) [74]	7.32 ± 0.93[a]	7.96 ± 0.39[a]	7.69 ± 0.30[a]	7.38 ± 0.68[a]
20.23	Isorhamnetin[e]	315	300 (100) [43]	2.66 ± 1.40[a]	3.80 ± 0.26[a]	3.42 ± 0.32[a]	4.01 ± 0.37[a]
	Anthocyanins	**(M^+)**					
8.80	Delphinidin-3-*O*-glucoside[f]	465	303 (100) [42]	14.37 ± 2.05[a]	16.06 ± 0.99[a]	16.01 ± 1.62[a]	14.60 ± 0.77[a]
9.52	Cyanidin-3-*O*-p-glucoside[f]	449	287 (100) [42]	3.72 ± 0.55[a]	3.57 ± 0.25[a]	4.06 ± 0.64[a]	3.53 ± 0.49[a]
10.83	Peonidin-3-*O*-glucoside[f]	463	301 (100) [42]	66.85 ± 3.38[a]	67.91 ± 2.15[a]	65.56 ± 1.74[a]	61.53 ± 2.73[b]
11.04	**Malvidin 3-*O*-glucoside[f]**	493	331 (100) [42]	1.84 ± 0.18[a]	2.34 ± 0.27[b]	2.33 ± 0.49[ab]	1.82 ± 0.11[a]
13.35	Petunidin-3-*O*-glucoside[f]	479	317 (100) [42]	3.73 ± 0.47[a]	4.09 ± 0.45[a]	3.98 ± 0.25[a]	3.69 ± 0.43[a]

14.31	Malvidin-3-*O*-acetylglucoside[f]	535	331 (100) [42]	22.44 ± 1.56^{a}	24.31 ± 1.69^{a}	22.09 ± 1.16^{a}	22.37 ± 2.41^{a}
16.23	Cyanidin-3-*O*-p-coumarylglucoside[f]	595	415 (20), 287 (100) [75,76]	2.91 ± 0.43^{a}	3.25 ± 0.43^{a}	3.17 ± 0.38^{a}	3.03 ± 0.49^{a}
17.22	Peonidin-3-*O*-(6-*O*-p-coumaroyl)glucoside[f]	609	301 (100) [42]	7.21 ± 0.74^{a}	8.02 ± 1.09^{a}	6.88 ± 0.93^{a}	7.71 ± 0.87^{a}
17.29	Malvidin-3-glucoside-p-*O*-coumarate[f]	639	331 (100) [42]	3.77 ± 0.53^{a}	3.94 ± 0.45^{a}	4.31 ± 0.87^{a}	3.29 ± 0.37^{a}
17.80	**Peonidin 3,5-*O*-diglucoside**[f]	625	463 (100), 301 (20) [42]	2.30 ± 0.31^{a}	2.59 ± 0.34^{ab}	2.26 ± 0.17^{a}	2.93 ± 0.38^{b}
18.44	**Malvidin-3-*O*-glucoside-4-vinylphenol**[f]	609	447 (100) [44]	4.37 ± 0.62^{a}	5.02 ± 0.67^{b}	3.94 ± 0.57^{a}	4.50 ± 0.47^{ab}

t_R – retention time, **$[M-H]^-$** - molecular ion at negative mode, **(M^+)** –molecular ion at positive mode. Different superscript lowercase letters in a row represent statistically significant differences between at least two wines bottled with different stoppers ($p < 0.05$), using Multiple t-tests in GraphPad prism. The results are expressed as the averages of 4 bottles × 3 replicates (n=12) ± the standard deviation. [a] Expressed in equivalents of gallic acid. [b] Expressed in equivalents of caffeic acid. [c] Expressed in equivalents of ellagic acid. [d] Expressed in equivalents of catechin. [e] Expressed in equivalents of quercetin. [f] Expressed in equivalents of cyanidin chloride.

Table S3. List of volatile compounds detected in the Douro red wine sealed with Natural Cork 1 and Micro A closures (stored for 35 months until analysis, in a horizontal position), using HS-SPME/GC×GC-ToFMS, including relevant chromatographic data used to assess compounds identification. The compounds marked in bold present statistically significant differences between the wines ($p < 0.05$).

^1t_R (s)[a]	^2t_R (s)[a]	Volatile compounds	CAS	RI $_{Calc}$[b]	RI $_{Lit}$[c]	Concentration (µg/L)	
						Natural Cork 1	Micro A
		Acids					
759	0.392	Acetic acid	64-19-7	1456	1452 [53]	145.365 ± 26.173[a]	152.259 ± 18.246[a]
906	0.424	Isobutyric acid	79-31-2	1575	1554 [77]	5.299 ± 1.149[a]	6.012 ± 0.505[a]
978	0.408	**Butyric acid**	107-92-6	1636	1624 [53]	3.230 ± 0.607[a]	2.737 ± 0.315[b]
990	0.376	2-Propenoic acid	79-10-7	1647	--	0.241 ± 0.035[a]	0.219 ± 0.033[a]
1023	0.424	Isovaleric acid	503-74-2	1675	1666 [53]	13.311 ± 0.975[a]	12.834 ± 1.387[a]
1101	0.416	Pentanoic acid	109-52-4	1744	1733 [53]	0.259 ± 0.038[a]	0.267 ± 0.043[a]
1326	0.456	Heptanoic acid	111-14-8	1952	1946 [77]	0.476 ± 0.076[a]	0.447 ± 0.063[a]
1455	0.472	**Octanoic Acid**	124-07-2	2065	2068 [78]	35.418 ± 3.621[a]	27.880 ± 3.061[b]
1527	0.432	**Nonanoic acid**	112-05-0	2128	2127 [77]	1.161 ± 0.500[a]	0.801 ± 0.170[b]
1578	0.432	**Decanoic acid**	334-48-5	2173	2250 [79]	4.330 ± 0.591[a]	2.555 ± 0.427[b]
		Alcohols					
		Aliphatics					
210	0.488	1-Propanol	71-23-8	1042	1045 [80]	35.583 ± 4.961[a]	33.360 ± 3.450[a]
294	0.600	3-Pentanol	584-02-1	1120	1111[81]	0.312 ± 0.014[a]	0.306 ± 0.025[a]
342	0.512	1-Butanol	71-36-3	1154	1145 [82]	10.816 ± 0.934[a]	11.070 ± 0.797[a]
435	0.592	Isoamyl alcohol	123-51-3	1221	1207 [83]	725.181 ± 176.412[a]	612.131 ± 155.194[a]
489	0.560	1-Pentanol	71-41-0	1257	1255 [84]	2.897 ± 0.356[a]	2.990 ± 0.317[a]
558	0.616	2-Methyl-1-pentanol	105-30-6	1305	--	0.097 ± 0.014[a]	0.097 ± 0.012[a]
576	0.600	Isohexyl alcohol	626-89-1	1318	1311 [85]	2.641 ± 0.149[a]	2.552 ± 0.068[a]
585	0.712	**2-Heptanol**	543-49-7	1324	1308 [86]	1.983 ± 0.100[a]	1.852 ± 0.089[b]

594	0.608	3-Methyl-1-pentanol	589-35-5	1331	1331 [87]	6.656 ± 0.437^{a}	6.421 ± 0.595^{a}
630	0.624	1-Hexanol	111-27-3	1358	1363 [88]	80.764 ± 9.942^{a}	79.471 ± 11.752^{a}
672	0.552	3-Hexen-1-ol	928-96-1	1387	1400 [89]	1.714 ± 0.070^{a}	1.680 ± 0.057^{a}
678	0.560	4-Methyl-3-penten-1-ol	763-89-3	1392	1385 [81]	0.251 ± 0.024^{a}	0.259 ± 0.032^{a}
720	0.776	**2-Octanol**	123-96-6	1424	1412 [90]	1.505 ± 0.081^{a}	1.412 ± 0.083^{b}
765	0.656	1-Heptanol	111-70-6	1460	1456 [91]	16.124 ± 0.885^{a}	15.763 ± 0.946^{a}
807	0.728	**2-Ethyl-1-hexanol**	104-76-7	1493	1493 [92]	10.317 ± 0.512^{a}	7.581 ± 0.325^{b}
816	0.600	4-Hepten-1-ol	20851-55-2	1501	1502 [81]	0.317 ± 0.026^{a}	0.307 ± 0.033^{a}
834	0.592	2-Hepten-1-ol	33467-76-4	1516	1491 [93]	0.488 ± 0.034^{a}	0.489 ± 0.054^{a}
843	0.84	**2-Nonanol**	628-99-9	1523	1522 [82]	2.586 ± 0.349^{a}	2.065 ± 0.130^{b}
891	0.712	1-Octanol	111-87-5	1563	1561 [82]	17.484 ± 1.000^{a}	16.082 ± 0.693^{a}
960	0.632	2-Octen-1-ol	18409-17-1	1621	1620 [92]	2.443 ± 0.153^{a}	2.397 ± 0.139^{a}
963	0.912	**2-Decanol**	1120-06-5	1624	1621 [94]	0.125 ± 0.015^{a}	0.104 ± 0.027^{b}
1011	0.76	**1-Nonanol**	143-08-8	1665	1668 [95]	7.671 ± 0.725^{a}	6.143 ± 0.349^{b}
1020	0.848	**2-Propyl-1-heptanol**	10042-59-8	1673	--	0.940 ± 0.096^{a}	0.774 ± 0.112^{b}
1047	0.696	4-Decen-1-ol	57074-37-0	1696	--	0.792 ± 0.089^{a}	0.734 ± 0.086^{a}
		Aromatics					
1122	0.552	**Dimethylbenzenemethanol**	617-94-7	1764	1776 [87]	0.109 ± 0.010^{a}	0.136 ± 0.015^{b}
1128	0.720	**Tetrahydroionol**	4361-23-3	1770	--	1.234 ± 0.088^{a}	1.082 ± 0.096^{b}
1248	0.456	Benzyl Alcohol	100-51-6	1882	1908 [96]	37.208 ± 3.565^{a}	36.895 ± 2.649^{a}
1281	0.608	Benzeneethanol	60-12-8	1913	1920 [53]	228.268 ± 72.131^{a}	251.891 ± 58.992^{a}
		Aldehydes					
		Aliphatics					
69	0.368	Acetaldehyde	75-07-0	685	718 [89]	54.626 ± 7.590^{a}	52.492 ± 5.862^{a}
114	0.624	3-Methylbutanal	590-86-3	916	922 [53]	0.872 ± 0.117^{a}	0.914 ± 0.087^{a}

255	0.952	Hexanal	66-25-1	1083	1087 [53]	0.909 ± 0.248[a]	0.861 ± 0.111[a]
540	1.200	Octanal	124-13-0	1293	1295 [97]	2.109 ± 1.374[a]	1.761 ± 0.485[a]
681	1.280	Nonanal	124-19-6	1394	1385 [98]	14.335 ± 12.185[a]	11.473 ± 2.898[a]
816	1.352	Decanal	112-31-2	1498	1499 [99]	14.997 ± 8.370[a]	13.715 ± 5.485[a]
		Aromatics					
846	0.592	**Benzaldehyde**	100-52-7	1526	1523 [83]	12.758 ± 0.994[a]	11.400 ± 0.713[b]
990	0.592	Phenylacetadehyde	122-78-1	1647	1652 [53]	4.387 ± 0.284[a]	4.148 ± 0.315[a]
		Aromatic hydrocarbons					
438	1.320	**Ethylmethylbenzene**	622-96-8	1224	1226 [100]	1.190 ± 0.179[a]	0.866 ± 0.120[b]
492	0.840	Styrene	100-42-5	1259	1260 [101]	8.236 ± 0.673[a]	7.618 ± 0.918[a]
561	1.464	**Butylbenzene**	104-51-8	1308	1304 [102]	0.244 ± 0.060[a]	0.139 ± 0.023[b]
582	1.408	**1-Ethyl-2,3-dimethylbenzene**	933-98-2	1323	1362 [103]	0.166 ± 0.010[a]	0.140 ± 0.027[b]
621	1.368	**1,4-Dimethyl-2-ethylbenzene**	1758-88-9	1351	1364 [80]	0.140 ± 0.015[a]	0.110 ± 0.025[b]
630	1.328	**1-Ethyl-2,4-dimethylbenzene**	874-41-9	1358	1373 [104]	0.161 ± 0.018[a]	0.132 ± 0.023[b]
738	1.048	**Isopropenyltoluene**	7399-49-7	1439	--	1.396 ± 0.064[a]	1.255 ± 0.124[b]
750	1.032	1-Ethenyl-4-ethylbenzene	3454-07-7	1448	--	0.683 ± 0.104[a]	0.677 ± 0.086[a]
861	1.184	**Mesitylethylene**	769-25-5	1539	--	0.319 ± 0.039[a]	0.162 ± 0.025[b]
900	0.816	**Vinyl styrene**	1515-78-2	1571	1572 [102]	0.109 ± 0.018[a]	0.126 ± 0.016[b]
		Dioxane compounds					
237	0.792	**2-Methyl-1,3-dioxane**	626-68-6	1071	1044 [81]	3.016 ± 1.235[a]	2.167 ± 0.117[b]
507	1.352	**1,3-Dioxane**	505-22-6	1270	--	0.492 ± 0.170[a]	0.358 ± 0.040[b]
		Esters					
105	0.528	Ethyl acetate	141-78-6	891	896 [53]	359.893 ± 102.165[a]	372.779 ± 74.583[a]
141	0.768	Ethyl propanoate	105-37-3	961	961 [105]	41.438 ± 7.379[a]	37.412 ± 6.516[a]
153	0.768	Propyl acetate	109-60-4	981	983 [106]	10.158 ± 2.209[a]	8.923 ± 0.849[a]

159	0.792	Methyl butonoate	623-42-7	991	989 [107]	0.183 ± 0.020[a]	0.210 ± 0.055[a]
204	1.096	Ethyl butyrate	105-54-4	1039	1020 [108]	65.969 ± 5.689[a]	64.038 ± 5.295[a]
222	1.384	Ethyl 2-methylbutanoate	7452-79-1	1057	1043 [109]	21.169 ± 1.632[a]	19.836 ± 1.797[a]
240	1.328	Ethyl 3-methylbutanoate	108-64-5	1072	1064 [109]	45.820 ± 2.858[a]	43.194 ± 3.916[a]
255	1.392	**Isobutyl propanoate**	540-42-1	1090	1083 [110]	0.260 ± 0.070[a]	0.209 ± 0.021[b]
261	1.808	**Isobutyl isobutyrate**	97-85-8	1096	1090 [26]	0.256 ± 0.020[a]	0.227 ± 0.029[b]
291	0.824	**Diethyl carbonate**	105-58-8	1118	1083 [111]	0.223 ± 0.012[a]	0.503 ± 0.046[b]
309	1.232	Isoamyl acetate	123-92-2	1131	1112 [82]	313.298 ± 62.195[a]	296.460 ± 48.426[a]
396	1.576	**Isoamyl propanoate**	105-68-0	1193	1183 [81]	3.239 ± 0.771[a]	2.596 ± 0.241[b]
402	1.968	**Isoamyl isobutanoate**	2050-01-3	1197	1214 [112]	3.239 ± 0.771[a]	2.596 ± 0.241[b]
465	1.560	Ethyl hexanoate	123-66-0	1241	1244 [113]	398.221 ± 88.086[a]	376.507 ± 95.016[a]
519	1.336	**Hexyl acetate**	142-92-7	1278	1279 [87]	13.413 ± 0.916[a]	11.974 ± 1.103[b]
519	2.136	**2-Methylbutyl 2-methylbutanoate**	2445-78-5	1277	1274 [81]	0.781 ± 0.080[a]	0.491 ± 0.080[b]
579	1.680	**Propyl hexanoate**	626-77-7	1321	1339 [114]	0.681 ± 0.133[a]	0.534 ± 0.071[a]
603	1.568	Ethyl heptanoate	106-30-9	1338	1332 [82]	15.748 ± 0.909[a]	15.882 ± 0.086[a]
621	0.600	Ethyl lactate	97-64-3	1348	1345 [83]	117.363 ± 16.071[a]	108.088 ± 9.220[a]
627	1.904	**Isobutyl hexanoate**	105-79-3	1356	1347 [85]	1.435 ± 0.067[a]	1.182 ± 0.179[b]
657	1.304	**Ethyl (E)-4-heptenoate**	54340-70-4	1377	1382 [110]	0.483 ± 0.046[a]	0.422 ± 0.044[b]
672	1.216	Ethyl 6-heptenoate	25118-23-4	1388	--	1.023 ± 0.620[a]	0.664 ± 0.076[a]
678	1.408	**Methyl octanoate**	111-11-5	1392	1394 [115]	13.913 ± 0.673[a]	10.531 ± 1.234[b]
738	1.864	**Ethyl octanoate**	106-32-1	1442	1434 [82]	1068.850 ± 173.225[a]	823.701 ± 169.095[b]
765	1.944	**Isopentyl hexanoate**	2198-61-0	1461	1464 [87]	12.355 ± 0.993[a]	10.177 ± 1.642[b]
771	0.648	Butyl lactate	138-22-7	1465	1520 [81]	2.503 ± 0.345[a]	2.433 ± 0.290[a]
801	1.296	**Ethyl 7-octenoate**	35194-38-8	1489	--	3.037 ± 0.820[a]	2.151 ± 0.204[b]
873	0.720	Ethyl 2-hydroxy-4-methylvalerate	10348-47-7	1548	1515 [81]	8.990 ± 2.142[a]	7.991 ± 0.725[a]

879	2.048	**Butyl caprylate**	589-75-3	1554	1601 [111]	1.481 ± 0.140[a]	1.060 ± 0.147[b]
882	1.312	**Ethyl (E)-2-octenoate**	7367-82-0	1556	1540 [116]	0.524 ± 0.060[a]	0.307 ± 0.051[b]
888	1.616	**4-tert-Butylcyclohexyl acetate**	32210-23-4	1561	--	0.117 ± 0.018[a]	0.266 ± 0.065[b]
903	0.696	Isoamyl lactate	19329-89-6	1573	1570 [82]	26.842 ± 4.823[a]	23.936 ± 1.505[a]
924	0.656	Diethyl malonate	105-53-3	1591	1580 [81]	0.388 ± 0.052[a]	0.350 ± 0.046[a]
984	1.808	**Ethyl caprate**	110-38-3	1643	1644 [112]	285.604 ± 61.586[a]	147.252 ± 18.678[b]
987	0.664	Ethyl methyl succinate	627-73-6	1644	1631 [117]	12.602 ± 0.895[a]	11.820 ± 1.178[a]
993	0.864	**Diethyl methylsuccinate**	4676-51-1	1650	--	0.920 ± 0.108[a]	0.829 ± 0.082[b]
1005	2.056	**Isoamyl octanoate**	2035-99-6	1661	1670 [87]	8.699 ± 0.911[a]	5.546 ± 0.883[b]
1014	0.776	**Ethyl benzoate**	93-89-0	1667	1644 [101]	2.601 ± 0.182[a]	2.206 ± 0.187[b]
1032	0.992	Diethyl succinate	123-25-1	1687	1684 [83]	438.222 ± 237.424[a]	466.620 ± 187.922[a]
1044	1.440	**Ethyl 9-decenoate**	67233-91-4	1694	1703 [87]	21.965 ± 1.595[a]	12.971 ± 1.718[b]
1089	0.672	**Benzyl acetate**	140-11-4	1734	1726 [81]	0.586 ± 0.062[a]	0.534 ± 0.038[b]
1101	0.448	Trimethylene acetate	628-66-0	1744	1660 [83]	1.815 ± 0.856[a]	1.518 ± 0.251[a]
1134	0.688	**Methyl salicylate**	119-36-8	1775	1778 [85]	7.842 ± 0.687[a]	6.630 ± 0.639[b]
1149	0.744	**Ethyl 2-phenylacetate**	101-97-3	1789	1783 [81]	4.486 ± 0.426[a]	4.039 ± 0.346[b]
1170	0.776	**Ethyl o-hydroxybenzoate**	118-61-6	1808	1828 [26]	2.035 ± 0.250[a]	1.571 ± 0.152[b]
1179	0.736	**2-Phenylethyl acetate**	103-45-7	1820	1820 [83]	41.605 ± 3.936[a]	37.884 ± 3.215[b]
1224	2.184	**3-Methylbutyl decanoate**	2306-91-4	1861	1859 [81]	0.517 ± 0.113[a]	0.280 ± 0.097[b]
1251	0.808	Ethyl 3-phenylpropanoate	2021-28-5	1886	1879 [81]	0.458 ± 0.062[a]	0.433 ± 0.089[a]
1485	0.712	**Ethyl 3-hydroxytridecanoate**	107141-15-1	2092	--	0.160 ± 0.029[a]	0.137 ± 0.025[b]
1503	0.600	**Ethyl 3-phenyl-2-propenoate**	103-36-6	2108	2108 [101]	0.101 ± 0.023[a]	0.081 ± 0.018[b]
1566	1.112	**Ethyl pentadecanoate**	41114-00-5	2163	2179 [87]	0.538 ± 0.334[a]	0.292 ± 0.073[b]
		Ethers					
657	0.544	3-Ethoxy-1-propanol	111-35-3	1377	1376 [83]	1.424 ± 0.873[a]	0.997 ± 0.066[a]

798	0.920	**Ethoxytoluene**	622-60-6	1486	--	0.102 ± 0.011[a]	0.069 ± 0.007[b]
933	1.120	**3-Methoxy-p-cymene**	1076-56-8	1598	1593 [118]	0.114 ± 0.065[a]	0.013 ± 0.002[b]
1110	0.664	**1,3-Dimethoxybenzene**	151-10-0	1753	--	0.079 ± 0.007[a]	0.067 ± 0.008[b]
1617	0.536	Benzyl ether	103-50-4	2208	--	1.036 ± 0.257[a]	0.989 ± 0.196[a]
		Furan derivatives					
81	0.416	Furan	110-00-9	813	802 [119]	0.430 ± 0.069[a]	0.461 ± 0.073[a]
198	1.104	**2-Ethyl-5-methylfuran**	1703-52-2	1033	1013 [120]	0.117 ± 0.018[a]	0.094 ± 0.018[b]
477	0.680	2-(Methoxymethyl)furan	13679-46-4	1248	1247 [121]	0.526 ± 0.074[a]	0.551 ± 0.040[a]
498	1.976	**2-Butyltetrahydrofuran**	1004-29-1	1264	--	0.656 ± 0.051[a]	0.586 ± 0.065[b]
783	0.504	Furfural	98-01-1	1474	1460 [83]	62.393 ± 8.098[a]	60.326 ± 4.427[a]
825	0.664	**Benzofuran**	271-89-6	1508	1489 [122]	2.077 ± 0.216[a]	1.675 ± 0.187[b]
831	0.536	1-(2-Furanyl)-Ethanone	1192-62-7	1513	1510 [121]	5.133 ± 0.763[a]	5.273 ± 0.178[a]
915	0.600	**1-(2-Furanyl)-1-propanone**	3194-15-8	1583	1571 [123]	0.302 ± 0.045[a]	0.494 ± 0.295[b]
915	0.536	5-Methyl-2-furfural	620-02-0	1583	1578 [121]	2.591 ± 0.687[a]	2.757 ± 0.244[a]
921	0.552	**Methyl 3-furancarboxylate**	1334-76-5	1588	--	0.508 ± 0.061[a]	0.603 ± 0.082[b]
930	0.760	**2-Methylbenzofuran**	4265-25-2	1596	1563 [120]	1.029 ± 0.111[a]	0.761 ± 0.080[b]
957	0.664	2-Furfurylfuran	1197-40-6	1619	1615 [121]	0.115 ± 0.026[a]	0.125 ± 0.013[a]
960	0.600	2-Acetyl-5-methylfuran	1193-79-9	1621	1608 [124]	0.125 ± 0.012[a]	0.121 ± 0.013[a]
972	0.608	Ethyl 2-Furoate	614-99-3	1631	1621 [82]	3.876 ± 0.255[a]	3.719 ± 0.181[a]
1020	0.424	**2-Furanmethanol**	98-00-0	1672	1669 [125]	24.093 ± 2.059[a]	25.591 ± 0.846[b]
1029	0.856	**4,7-Dimethylbenzofuran**	28715-26-6	1680	--	0.166 ± 0.012[a]	0.109 ± 0.012[b]
1032	0.472	5-Methyl-2(5H)-furanone	591-11-7	1683	1658 [120]	0.203 ± 0.045[a]	0.239 ± 0.049[a]
1068	0.456	Itaconic anhydride	2170-03-8	1714	1680 [102]	0.940 ± 0.295[a]	0.757 ± 0.161[a]
1083	0.544	**5-Ethoxydihydro-2(3H)-furanone**	932-85-4	1728	--	0.248 ± 0.030[a]	0.269 ± 0.016[b]
1119	0.432	2(5H)-Furanone	497-23-4	1761	1767 [126]	0.378 ± 0.028[a]	0.389 ± 0.026[a]

1305	0.432	Methylsuccinic anhydride	4100-80-5	1934	--	0.413 ± 0.138[a]	0.318 ± 0.098[a]
1641	0.352	**2-Furancarboxylic acid**	88-14-2	2228	--	1.895 ± 0.473[a]	1.511 ± 0.199[b]
		Ketones					
		Aliphatics					
237	0.624	2,3-Pentanedione	600-14-6	1071	1068 [53]	0.420 ± 0.137[a]	0.411 ± 0.029[a]
318	0.656	**(E)-3-Penten-2-one**	3102-33-8	1137	1121 [85]	0.560 ± 0.136[a]	0.438 ± 0.031[b]
489	1.280	**3-Octanone**	106-68-3	1257	1261 [127]	0.314 ± 0.053[a]	0.361 ± 0.057[b]
675	1.232	2-Nonanone	821-55-6	1390	1387 [82]	0.632 ± 0.269[a]	0.690 ± 0.077[a]
870	1.216	**3-Hexen-2-one**	763-93-9	1546	--	0.206 ± 0.029[a]	0.145 ± 0.027[b]
936	1.376	2-Undecanone	112-12-9	1601	1599 [128]	0.332 ± 0.135[a]	0.333 ± 0.112[a]
		Aromatics					
1080	0.696	**Propiophenone**	93-55-0	1725	--	0.056 ± 0.007[a]	0.063 ± 0.004[b]
1326	0.680	Benzalacetone	1896-62-4	1952	--	0.249 ± 0.017[a]	0.273 ± 0.055[a]
		Cyclics					
564	1.320	2,2,6-Trimethylcyclohexanone	2408-37-9	1310	1312 [129]	0.616 ± 0.044[a]	0.582 ± 0.074[a]
927	0.472	**2-Cyclopentene-1,4-dione**	930-60-9	1593	1576 [130]	0.418 ± 0.061[a]	0.515 ± 0.120[b]
		Lactones					
1404	0.544	Dehydromevalonic lactone	2381-87-5	2021	--	0.068 ± 0.012[a]	0.060 ± 0.006[a]
1413	0.736	γ-Nonalactone	104-61-0	2029	1998 [113]	0.683 ± 0.085[a]	0.626 ± 0.089[a]
1425	0.424	Pantolactone	599-04-2	2039	2033 [131]	0.437 ± 0.099[a]	0.398 ± 0.048[a]
		Naphthalene compounds					
1092	0.736	**Naphthalene**	91-20-3	1737	1740 [103]	0.410 ± 0.031[a]	0.445 ± 0.044[b]
1248	0.784	**1-Methylnaphthalene**	90-12-0	1883	1875 [132]	0.063 ± 0.007[a]	0.080 ± 0.015[b]
1368	0.968	1,8-Dimethylnaphthalene	569-41-5	1989	1989 [133]	0.140 ± 0.018[a]	0.137 ± 0.041[a]
1524	0.680	**1,4,5-Trimethylnaphthalene**	2131-41-1	2126	--	0.055 ± 0.009[a]	0.046 ± 0.009[b]

1551	0.704	**Cadelene**	483-78-3	2150	2200 [134]	0.673 ± 0.096[a]	0.357 ± 0.068[b]
		Norisoprenoids					
984	0.912	**Safranal**	116-26-7	1642	--	0.325 ± 0.041[a]	0.271 ± 0.022[b]
1095	1.168	**TDN**	30364-38-6	1740	1724 [133]	5.328 ± 0.646[a]	2.538 ± 0.428[b]
1179	1.040	**β-Damascenone**	23726-93-4	1817	1830 [53]	1.687 ± 0.246[a]	1.380 ± 0.132[b]
		Phenol-derivatives					
1020	0.824	**Estragole**	140-67-0	1673	1671 [135]	0.094 ± 0.022[a]	0.061 ± 0.011[b]
1164	0.624	**4-Hydroxyacetophenone**	99-93-4	1803	1786 [103]	0.039 ± 0.004[a]	0.035 ± 0.003[b]
1332	0.560	Creosol	93-51-6	1958	1956 [136]	0.212 ± 0.020[a]	0.210 ± 0.020[a]
1395	0.416	Phenol	108-95-2	2013	2008 [53]	1.257 ± 0.098[a]	1.261 ± 0.179[a]
1395	0.448	2-Methylphenol	95-48-7	2013	2012 [94]	0.270 ± 0.023[a]	0.256 ± 0.028[a]
1422	0.592	**4-Ethylguaiacol**	2785-89-9	2037	2039 [83]	0.030 ± 0.005[a]	0.025 ± 0.003[b]
1593	0.488	**2,4-Di-tert-butylphenol**	96-76-4	2186	2280 [130]	0.326 ± 0.033[a]	0.258 ± 0.045[b]
		Sulphur compounds					
219	0.664	Methylthiolacetate	1534-08-3	1054	1057 [119]	0.640 ± 0.034[a]	0.626 ± 0.064[a]
243	0.752	Dimethyl disulfide	624-92-0	1077	1063 [137]	0.110 ± 0.020[a]	0.115 ± 0.037[a]
765	0.568	**Methional**	3268-49-3	1460	1480 [127]	0.012 ± 0.002[a]	0.023 ± 0.011[b]
849	0.632	**2-Methyl-3-thiolanone**	13679-85-1	1528	1525 [130]	1.450 ± 0.402[a]	1.779 ± 0.103[b]
1050	0.52	2-Thiophenecarboxaldehyde	98-03-3	1698	1684 [81]	0.428 ± 0.058[a]	0.413 ± 0.030[a]
1074	0.48	3-(Methylthio)-1-propanol	505-10-2	1720	1719 [83]	15.152 ± 1.199[a]	15.492 ± 1.339[a]
		Terpenic compounds					
276	2.432	**Linalool-3,7-oxide**	7392-19-0	1108	1112 [132]	3.060 ± 0.462[a]	2.684 ± 0.258[b]
396	2.208	**Limonene**	5989-54-8	1193	1191 [135]	6.095 ± 1.389[a]	3.938 ± 0.965[b]
405	2.152	Eucalyptol	470-82-6	1199	1195 [135]	0.269 ± 0.033[a]	0.338 ± 0.150[a]
468	2.032	**γ-Terpinene**	99-85-4	1243	1274 [89]	0.653 ± 0.116[a]	0.492 ± 0.089[b]

516	2.024	**α-Terpinolene**	586-62-9	1276	1315 [89]	1.312 ± 0.166[a]	1.115 ± 0.144[b]
621	1.608	**Rose oxide**	16409-43-1	1351	1339 [138]	0.386 ± 0.027[a]	0.314 ± 0.046[b]
777	1.224	**Nerol oxide**	1786-08-9	1470	1468.5 [139]	1.885 ± 0.215[a]	1.662 ± 0.153[b]
822	1.096	**Camphor**	76-22-2	1506	1458 [140]	0.249 ± 0.176	--#
879	0.792	Linalool	78-70-6	1553	1526 [135]	15.914 ± 0.898[a]	15.986 ± 0.738[a]
915	0.800	Fenchol	1632-73-1	1583	1574 [84]	0.078 ± 0.033[a]	0.076 ± 0.111[a]
936	0.936	**Terpinen-4-ol**	562-74-3	1601	1605 [101]	1.029 ± 0.061[a]	0.949 ± 0.059[b]
1050	0.776	**α-Terpineol**	98-55-5	1698	1686 [141]	9.777 ± 0799[a]	9.061 ± 0.548[b]
1071	2.104	**α-Muurolene**	31983-22-9	1718	--	2.758 ± 0.471[a]	1.295 ± 0.240[b]
1167	0.696	Nerol	106-25-2	1805	1803 [142]	0.229 ± 0.021[a]	0.224 ± 0.029[a]
1188	1.616	**Calamenene**	483-77-2	1826	1832 [143]	0.424 ± 0.064[a]	0.223 ± 0.069[b]
1263	1.392	**α-Calacorene**	21391-99-1	1898	1916 [144]	0.113 ± 0.014[a]	0.036 ± 0.008[b]
1434	1.048	**Nerolidol**	7212-44-4	2047	2046 [145]	0.709 ± 0.068[a]	0.478 ± 0.121[b]
		Other Compounds					
525	0.592	Acetic anhydride	108-24-7	1282	--	1.681 ± 0.296[a]	1.558 ± 0.128[a]
639	1.088	**Indane**	496-11-7	1364	1365 [103]	2.077 ± 0.216[a]	0.192 ± 0.025[b]
768	1.328	**3-Methylene-tridecane**	19780-34-8	1463	--	5.541 ± 0.914[a]	4.501 ± 1.024[b]
828	0.864	**Ethyl sorbate**	2396-84-1	1511	1501 [136]	0.199 ± 0.015[a]	0.171 ± 0.028[b]
945	0.560	Benzonitrile	100-47-0	1608	1583 [146]	0.259 ± 0.035[a]	0.269 ± 0.032[a]
975	1.112	**1,2,3-Trimethylindene**	4773-83-5	1634	--	0.387 ± 0.050[a]	0.173 ± 0.027[b]
1098	1.080	4-Phenylcyclohexene	4994-16-5	1742	--	0.352 ± 0.033[a]	0.360 ± 0.062[a]
1128	0.896	Dibutylformamide	761-65-9	1770	1767 [103]	0.233 ± 0.069[a]	0.212 ± 0.042[a]
1167	0.504	**Tetrahydro-2H-pyran-2-one**	542-28-9	1805	1780 [101]	0.210 ± 0.024[a]	0.183 ± 0.017[b]

[a] Retention time for first (^1t_R) and second (^2t_R) dimensions in seconds. [b] RI: Linear Retention Index obtained through the modulated chromatogram. [c] RI: Linear Retention Index reported in the literature for DB-FFAP column or equivalents. The results are expressed as the averages of 4 bottles × 3 replicates (*n* = *12*) ± the standard deviation. The

concentrations are expressed as equivalents of 3-Octanol. Different superscript lowercase letters in a row represent statistically significant differences between wines bottled with different stoppers at $p < 0.05$, using Two-Way ANOVA (followed by Tukey´s multiple comparison test) in GraphPad prism. # – not detected.

Table S4. List of volatile compounds detected in the Austria red wine sealed with Natural Cork 2, Micro A, Micro E and Screw Cap stoppers (stored for 5 months until analysis, in a horizontal position), using HS-SPME/GC×GC-ToFMS, including relevant chromatographic data used to assess compounds identification. The compounds marked in bold present statistically significant differences at least between two wines ($p < 0.05$).

$^{1}t_R$ (s)[a]	$^{2}t_R$ (s)[a]	Volatile compounds	CAS	RI $_{Calc}$[b]	RI $_{Lit}$[c]	Concentration (μg/L)			
						Natural Cork 2	Micro A	Micro E	Screw cap
		Acids							
762	0.384	**Acetic acid**	64-19-7	1456	1452 [53]	159.646 ± 17.182[a]	134.835 ± 11.714[b]	143.600 ± 12.881[ab]	145.958 ± 11.013[ab]
906	0.424	Isobutyric acid	79-31-2	1573	1554 [77]	6.478 ± 0.836[a]	6.931 ± 1.124[a]	6.331 ± 1.491[a]	6.471 ± 1.107[a]
978	0.408	**Butyric acid**	107-92-6	1636	1624 [53]	4.518 ± 0.623[ab]	4.978 ± 0.772[a]	4.486 ± 0.450[ab]	4.256 ± 0.445[b]
990	0.376	**2-Propenoic acid**	79-10-7	1647	--	0.209 ± 0.068[a]	0.272 ± 0.045[b]	0.262 ± 0.039[ab]	0.237 ± 0.035[ab]
1020	0.440	Isovaleric acid	503-74-2	1673	1666 [53]	19.265 ± 2.396[a]	19.702 ± 2.393[a]	18.383 ± 1.999[a]	19.022 ± 1.750[a]
1098	0.432	**Pentanoic acid**	109-52-4	1742	1733 [53]	0.267 ± 0.049[ab]	0.294 ± 0.033[a]	0.263 ± 0.046[ab]	0.238 ± 0.020[b]
1212	0.464	Hexanoic acid	142-62-1	1851	1839 [53]	36.050 ± 5.442[a]	34.370 ± 3.345[a]	31.671 ± 3.804[a]	34.056 ± 3.059[a]
1326	0.456	**Heptanoic acid**	111-14-8	1950	1946 [77]	0.570 ± 0.169[ab]	0.571 ± 0.093[a]	0.508 ± 0.114[ab]	0.439 ± 0.045[b]
1449	0.504	**Octanoic acid**	124-07-2	2064	2068 [78]	38.721 ± 8.362[ab]	33.491 ± 3.234[ab]	32.147 ± 3.738[a]	37.630 ± 3.012[b]
1578	0.472	**Decanoic acid**	334-48-5	2171	2250 [79]	4.784 ± 1.407[ac]	3.690 ± 0.576[ab]	3.488 ± 0.776[b]	4.286 ± 0.351[c]
		Alcohols							
		Aliphatics							
207	0.488	**1-Propanol**	71-23-8	1042	1045 [80]	23.844 ± 2.180[a]	19.322 ± 1.360[b]	21.219 ± 2.528[ab]	21.958 ± 0.967[a]
291	0.632	**3-Pentanol**	584-02-1	1119	1111 [81]	0.209 ± 0.018[ab]	0.224 ± 0.018[a]	0.191 ± 0.012[bd]	0.191 ± 0.012[cd]
345	0.520	**1-Butanol**	71-36-3	1151	1145 [82]	7.711 ± 0.666[ab]	7.741 ± 0.351[a]	7.256 ± 0.477[b]	7.250 ± 0.417[ab]
360	0.544	**1-Peten-3-ol**	616-25-1	1166	1166 [147]	0.660 ± 0.055[ab]	0.715 ± 0.048[a]	0.642 ± 0.045[bd]	0.609 ± 0.034[cd]
429	0.592	Isoamyl alcohol	123-51-3	1216	1207 [83]	827.849 ± 150.601[a]	674.538 ± 156.244[a]	727.312 ± 176.971[a]	666.072 ± 172.161[a]
486	0.576	**1-Pentanol**	71-41-0	1255	1255 [84]	1.552 ± 0.144[a]	1.898 ± 0.281[b]	1.699 ± 0.311[ab]	1.709 ± 0.128[b]
573	0.616	**Isohexyl alcohol**	626-89-1	1316	1311 [85]	3.083 ± 0.211[ab]	3.353 ± 0.178[a]	3.247 ± 0.200[ab]	3.100 ± 0.150[b]
582	0.744	**2-Heptanol**	543-49-7	1322	1308 [86]	1.207 ± 0.091[a]	1.431 ± 0.088[b]	1.420 ± 0.072[bc]	1.310 ± 0.082[ac]
591	0.624	**3-Methyl-1-pentanol**	589-35-5	1329	1331 [87]	5.199 ± 0.380[a]	5.812 ± 0.321[b]	5.618 ± 0.314[ab]	5.374 ± 0.268[a]
627	0.640	1-Hexanol	111-27-3	1358	1363 [88]	81.896 ± 8.666[a]	73.820 ± 8.918[a]	76.804 ± 7.437[a]	79.435 ± 8.739[a]
669	0.576	**3-Hexen-1-ol**	928-96-1	1385	1400 [89]	2.266 ± 0.171[a]	2.592 ± 0.163[b]	2.474 ± 0.211[ab]	2.321 ± 0.092[a]
711	0.576	**(E)-2-Hexen-1-ol**	928-95-0	1417	1407 [148]	0.744 ± 0.047[a]	0.881 ± 0.060[b]	0.831 ± 0.082[ab]	0.758 ± 0.036[a]

717	0.808	**2-Octanol**	123-96-6	1422	1412 [90]	0.363 ± 0.019^{a}	0.430 ± 0.024^{b}	0.409 ± 0.038^{bc}	0.375 ± 0.014^{ac}
756	0.704	**7-Octen-4-ol**	53907-72-5	1453	1453 [149]	2.158 ± 0.144^{a}	2.437 ± 0.154^{b}	2.377 ± 0.132^{b}	2.342 ± 0.089^{b}
762	0.672	**1-Heptanol**	111-70-6	1458	1456 [91]	9.342 ± 0.568^{ab}	10.039 ± 0.521^{a}	9.400 ± 0.356^{b}	9.944 ± 0.493^{ab}
804	0.752	**2-Ethyl-1-hexanol**	104-76-7	1491	1493 [92]	1.138 ± 0.068^{a}	1.323 ± 0.086^{b}	1.267 ± 0.121^{b}	1.133 ± 0.038^{a}
825	0.592	**4-Hepten-1-ol**	20851-55-2	1506	1502 [81]	0.381 ± 0.030^{a}	0.446 ± 0.030^{b}	0.424 ± 0.029^{ab}	0.392 ± 0.020^{ab}
840	0.888	**2-Nonanol**	628-99-9	1523	1522 [82]	1.773 ± 0.113^{a}	1.971 ± 0.135^{b}	1.827 ± 0.194^{ab}	1.896 ± 0.071^{ab}
888	0.736	**1-Octanol**	111-87-5	1561	1561 [82]	6.782 ± 0.394^{a}	7.694 ± 0.466^{b}	6.812 ± 0.537^{a}	7.205 ± 0.304^{ab}
951	0.728	**Hotrienol**	29957-43-5	1613	1613 [150]	1.283 ± 0.073^{a}	1.592 ± 0.116^{b}	1.417 ± 0.123^{ac}	1.385 ± 0.070^{c}
960	0.632	**2-Octen-1-ol**	18409-17-1	1621	1620 [92]	0.453 ± 0.054^{a}	0.516 ± 0.064^{b}	0.450 ± 0.058^{a}	0.453 ± 0.052^{ab}
1011	0.800	**1-Nonanol**	143-08-8	1662	1668 [95]	4.240 ± 0.491^{ab}	4.232 ± 0.491^{ab}	3.867 ± 0.423^{a}	4.480 ± 0.216^{b}
1077	0.960	**2-Undecanol**	1653-30-1	1723	1717 [151]	0.168 ± 0.033^{a}	0.132 ± 0.014^{b}	0.133 ± 0.024^{b}	0.192 ± 0.024^{a}
		Aromatics							
1119	0.568	**Dimethylbenzenemethanol**	617-94-7	1761	1776 [87]	0.174 ± 0.034^{ab}	0.193 ± 0.034^{b}	0.154 ± 0.036^{a}	0.147 ± 0.029^{a}
1128	0.720	**Tetrahydroionol**	4361-23-3	1770	--	1.478 ± 0.297^{ab}	1.507 ± 0.159^{a}	1.390 ± 0.193^{ab}	1.304 ± 0.079^{b}
1242	0.472	Benzyl alcohol	100-51-6	1877	1908 [96]	10.530 ± 1.617^{a}	10.798 ± 1.200^{a}	9.783 ± 1.186^{a}	10.172 ± 0.697^{a}
1278	0.536	**Benzeneethanol**	60-12-8	1913	1920 [53]	349.702 ± 83.525^{a}	269.819 ± 67.532^{ab}	236.844 ± 37.349^{b}	250.998 ± 55.664^{b}
		Cyclics							
1095	0.792	**4-tert-Butylcyclohexanol**	98-52-2	1742	--	0.096 ± 0.023^{ac}	0.110 ± 0.014^{a}	1.424 ± 0.151^{b}	0.083 ± 0.008^{c}
		Aldehydes							
		Aliphatics							
69	0.368	**Acetaldehyde**	75-07-0	685	718 [89]	34.908 ± 4.164^{a}	29.020 ± 3.099^{b}	29.169 ± 7.034^{ab}	30.917 ± 4.229^{ab}
111	0.616	**2-Methylbutanal**	96-17-3	911	915 [53]	0.652 ± 0.138^{a}	0.499 ± 0.087^{b}	0.583 ± 0.126^{ab}	0.616 ± 0.110^{a}
114	0.608	**3-Methylbutanal**	590-86-3	916	922 [53]	1.885 ± 0.908^{ab}	1.544 ± 0.287^{a}	1.188 ± 0.235^{b}	1.048 ± 0.213^{b}
252	0.984	**Hexanal**	66-25-1	1086	1087 [53]	0.896 ± 0.220^{a}	0.860 ± 0.167^{a}	0.749 ± 0.147^{ab}	0.663 ± 0.102^{b}
537	1.192	**Octanal**	124-13-0	1288	1295 [97]	2.327 ± 1.006^{a}	3.572 ± 1.136^{b}	2.942 ± 0.888^{c}	2.307 ± 0.507^{ac}
681	1.280	**Nonanal**	124-19-6	1394	1385 [98]	14.015 ± 8.722^{ab}	18.634 ± 6.490^{a}	16.255 ± 5.645^{ab}	11.964 ± 1.929^{b}
816	1.352	**Decanal**	112-31-2	1498	1499 [99]	9.721 ± 3.638^{ab}	10.990 ± 4.391^{a}	10.146 ± 4.356^{ab}	7.188 ± 1.534^{b}
		Aromatics							
846	0.592	**Benzaldehyde**	100-52-7	1526	1523 [83]	9.642 ± 1.094^{a}	8.761 ± 0.293^{a}	8.660 ± 0.638^{a}	6.609 ± 0.355^{b}
987	0.608	Phenylacetadehyde	122-78-1	1644	1652 [53]	4.363 ± 0.522^{a}	4.146 ± 0.433^{a}	4.047 ± 0.391^{a}	4.117 ± 0.233^{a}
1026	0.592	**Hidroxybenzaldehyde**	90-02-8	1677	1674 [103]	0.189 ± 0.034^{a}	0.171 ± 0.014^{a}	0.164 ± 0.018^{a}	0.121 ± 0.007^{b}
		Aromatic hydrocarbons							

312	1.216	**Xylene**	108-38-3	1133	1142 [119]	0.698 ± 0.204[a]	0.538 ± 0.057[a]	0.473 ± 0.039[b]	0.433 ± 0.038[b]
438	1.320	**Ethylmethylbenzene**	622-96-8	1222	1226 [98]	0.427 ± 0.199[a]	0.266 ± 0.035[ab]	0.242 ± 0.032[b]	0.256 ± 0.025[ab]
738	1.048	**Isopropenyltoluene**	7399-49-7	1439	--	1.546 ± 0.857[a]	0.768 ± 0.081[b]	0.716 ± 0.063[b]	0.746 ± 0.079[b]
		Esters							
84	0.440	**Methyl acetate**	79-20-9	824	825 [152]	11.646 ± 0.998[a]	9.823 ± 0.950[b]	10.329 ± 1.529[ab]	10.020 ± 0.673[b]
141	0.792	**Ethyl propanoate**	105-37-3	961	961 [105]	26.003 ± 2.808[a]	21.454 ± 1.677[b]	21.493 ± 1.282[b]	23.393 ± 1.259[a]
144	0.976	**Ethyl isobutyrate**	97-62-1	967	967 [108]	55.835 ± 3.360[a]	40.909 ± 5.107[b]	47.079 ± 5.046[b]	55.958 ± 3.727[a]
153	0.768	**Propyl acetate**	109-60-4	976	983 [106]	4.294 ± 0.731[ab]	3.844 ± 0.273[a]	4.136 ± 0.219[b]	4.254 ± 0.395[ab]
159	0.824	Methyl butonoate	623-42-7	996	989 [107]	0.175 ± 0.048[a]	0.155 ± 0.039[a]	0.167 ± 0.034[a]	0.148 ± 0.029[a]
180	1.040	**Isobutyl acetate**	110-19-0	1016	1013 [98]	21.090 ± 1.542[a]	19.169 ± 1.383[b]	20.680 ± 1.331[ab]	21.315 ± 1.398[a]
201	1.128	**Ethyl butyrate**	105-54-4	1036	1020 [108]	62.429 ± 5.336[a]	52.423 ± 4.329[b]	55.254 ± 3.924[bc]	58.074 ± 3.133[ac]
219	1.448	**Ethyl 2-methylbutanoate**	7452-79-1	1054	1043 [109]	24.085 ± 1.620[a]	21.292 ± 1.291[b]	22.346 ± 1.866[ab]	26.072 ± 1.646[c]
237	1.376	**Ethyl 3-methylbutanoate**	108-64-5	1072	1064 [109]	45.264 ± 2.793[a]	39.239 ± 2.568[b]	39.708 ± 3.286[b]	47.027 ± 3.044[a]
243	1.096	**Butyl acetate**	123-86-4	1078	1075 [153]	0.527 ± 0.042[ab]	0.558 ± 0.051[a]	0.508 ± 0.034[b]	0.491 ± 0.036[b]
249	1.472	**Isobutyl propanoate**	540-42-1	1084	1083 [110]	0.115 ± 0.011[a]	0.118 ± 0.013[ab]	0.106 ± 0.005[b]	0.115 ± 0.011[ab]
288	0.848	**Diethyl carbonate**	105-58-8	1116	1083 [111]	0.213 ± 0.019[a]	0.215 ± 0.021[a]	0.190 ± 0.015[b]	0.171 ± 0.014[c]
303	1.280	**Isoamyl acetate**	123-92-2	1131	1112 [82]	245.900 ± 30.066[a]	209.129 ± 27.717[b]	195.955 ± 27.092[b]	210.058 ± 27.873[b]
321	1.408	**Ethyl pentanoate**	539-82-2	1139	1134 [113]	2.300 ± 0.137[ab]	2.361 ± 0.146[a]	2.113 ± 0.149[b]	2.199 ± 0.126[ab]
363	0.936	**Ethyl 2-butenoate**	10544-63-5	1169	1165 [92]	1.561 ± 0.100[ab]	1.671 ± 0.096[a]	1.523 ± 0.080[b]	1.474 ± 0.090[b]
393	1.288	**Methyl hexanoate**	106-70-7	1190	1189 [115]	2.344 ± 0.129[ab]	2.427 ± 0.180[a]	2.175 ± 0.186[b]	2.235 ± 0.142[ab]
396	1.576	Isoamyl propanoate	105-68-0	1193	1183 [83]	1.441 ± 0.111[a]	1.494 ± 0.074[a]	1.476 ± 0.165[a]	1.542 ± 0.134[a]
465	1.560	Ethyl hexanoate	123-66-0	1241	1244 [113]	374.064 ± 81.606[a]	311.251 ± 59.336[a]	343.329 ± 54.832[a]	354.280 ± 44.426[a]
516	1.392	**Hexyl acetate**	142-92-7	1276	1279 [87]	5.163 ± 0.234[a]	5.193 ± 0.299[a]	5.132 ± 0.453[ab]	5.635 ± 0.366[b]
603	1.568	**Ethyl heptanoate**	106-30-9	1338	1332 [82]	13.396 ± 0.993[a]	11.546 ± 0.767[b]	11.209 ± 0.846[b]	13.620 ± 0.828[a]
624	2.024	**Isobutyl hexanoate**	105-79-3	1354	1347 [85]	1.066 ± 0.063[a]	0.893 ± 0.059[b]	0.904 ± 0.089[b]	1.123 ± 0.115[a]
657	1.304	**Ethyl (E)-4-heptenoate**	54340-70-4	1377	1382 [110]	0.321 ± 0.039[ac]	0.274 ± 0.020[bd]	0.266 ± 0.034[ab]	0.308 ± 0.036[cd]
678	1.400	**Methyl octanoate**	111-11-5	1392	1394 [115]	11.112 ± 0.607[a]	9.411 ± 0.455[b]	9.248 ± 0.699[b]	11.969 ± 0.645[c]
723	0.720	Ethyl 2-hydroxyisovalerate	2441-06-7	1427	1427 [139]	0.744 ± 0.054[a]	0.829 ± 0.082[a]	0.805 ± 0.073[a]	0.757 ± 0.042[a]
738	1.776	**Ethyl octanoate**	106-32-1	1436	1434 [82]	826.627 ± 121.210[a]	604.503 ± 133.217[b]	661.334 ± 143.318[ab]	713.330 ± 207.596[ab]
765	2.024	**Isopentyl hexanoate**	2198-61-0	1461	1464 [87]	8.777 ± 0.745[a]	6.940 ± 0.422[b]	7.216 ± 0.849[b]	9.283 ± 0.559[a]
870	0.736	Ethyl 2-hydroxy-4-methylvalerate	10348-47-7	1546	1515 [81]	8.713 ± 0.840[a]	9.658 ± 0.940[a]	8.695 ± 0.843[a]	8.900 ± 0.654[a]

879	2.152	**Butyl caprylate**	589-75-3	1554	1601 [111]	1.022 ± 0.140[a]	0.753 ± 0.071[b]	0.761 ± 0.087[b]	0.971 ± 0.071[a]
885	1.704	**4-tert-Butylcyclohexyl acetate**	32210-23-4	1561	--	0.132 ± 0.034[ab]	0.156 ± 0.018[a]	0.112 ± 0.017[b]	0.146 ± 0.016[a]
903	0.736	**Isoamyl lactate**	19329-89-6	1571	1570 [82]	13.214 ± 0.935[a]	14.804 ± 1.243[b]	13.502 ± 1.248[ab]	13.940 ± 1.057[ab]
921	0.680	Diethyl malonate	105-53-3	1588	1580 [81]	0.716 ± 0.072[a]	0.788 ± 0.073[a]	0.727 ± 0.078[a]	0.713 ± 0.050[a]
984	0.688	Ethyl methyl succinate	627-73-6	1644	1631 [117]	6.519 ± 0.429[a]	7.230 ± 0.850[a]	6.566 ± 0.732[a]	6.836 ± 0.575[a]
993	0.864	Diethyl methylsuccinate	4676-51-1	1650	--	0.437 ± 0.097[a]	0.413 ± 0.042[a]	0.384 ± 0.044[a]	0.403 ± 0.029[a]
1014	0.808	**Ethyl benzoate**	93-89-0	1667	1644 [101]	1.648 ± 0.219[a]	1.426 ± 0.099[b]	1.361 ± 0.085[b]	1.689 ± 0.109[a]
1086	0.696	Benzyl acetate	140-11-4	1731	1726 [81]	0.147 ± 0.025[a]	0.147 ± 0.017[a]	0.132 ± 0.011[a]	0.133 ± 0.011[a]
1098	0.464	Trimethylene acetate	628-66-0	1742	1660 [83]	0.532 ± 0.122[a]	0.566 ± 0.139[a]	0.469 ± 0.134[a]	0.500 ± 0.114[a]
1134	0.688	**Methyl salicylate**	119-36-8	1772	1778 [85]	0.913 ± 0.166[a]	0.803 ± 0.078[ab]	0.765 ± 0.071[b]	0.799 ± 0.053[ab]
1143	0.824	Diethyl glutarate	818-38-2	1783	1780 [81]	1.451 ± 0.254[a]	1.537 ± 0.195[a]	1.383 ± 0.201[a]	1.439 ± 0.115[a]
1146	0.768	**Ethyl 2-phenylacetate**	101-97-3	1786	1783 [81]	16.910 ± 2.894[ab]	15.782 ± 1.143[ab]	14.757 ± 1.202[a]	16.948 ± 0.740[b]
1167	0.816	**Ethyl o-hydroxybenzoate**	118-61-6	1806	1828 [26]	0.198 ± 0.044[a]	0.154 ± 0.015[b]	0.150 ± 0.015[b]	0.187 ± 0.015[a]
1179	0.760	2-Phenylethyl acetate	103-45-7	1817	1820 [83]	18.848 ± 3.770[a]	16.263 ± 1.278[a]	15.875 ± 1.347[a]	17.028 ± 1.078[a]
1251	0.808	Ethyl 3-phenylpropanoate	2021-28-5	1886	1879 [81]	0.684 ± 0.182[a]	0.560 ± 0.058[a]	0.545 ± 0.058[a]	0.596 ± 0.049[a]
1434	0.568	Diethyl hydroxybutanoate	626-11-9	2047	2062 [154]	1.409 ± 0.343[a]	1.427 ± 0.245[a]	1.272 ± 0.300[a]	1.422 ± 0.195[a]
1500	0.624	**Ethyl 3-phenyl-2-propenoate**	103-36-6	2105	2108 [101]	0.083 ± 0.012[a]	0.068 ± 0.012[b]	0.069 ± 0.007[b]	0.081 ± 0.007[a]
		Ethers							
612	0.752	**Anisole**	100-66-3	1344	1341 [26]	0.405 ± 0.036[a]	0.361 ± 0.023[b]	0.348 ± 0.022[b]	0.349 ± 0.038[b]
654	0.560	3-Ethoxy-1-propanol	111-35-3	1374	1376 [83]	1.022 ± 0.145[a]	1.156 ± 0.164[a]	1.126 ± 0.153[a]	1.030 ± 0.083[a]
1614	0.560	Benzyl ether	103-50-4	2205	--	1.973 ± 0.540[a]	1.962 ± 0.364[a]	1.770 ± 0.316[a]	1.832 ± 0.478[a]
		Furan derivatives							
96	0.576	**Tetrahydrofuran**	109-99-9	869	857 [155]	0.360 ± 0.101[a]	0.364 ± 0.081[a]	0.294 ± 0.063[ab]	0.271 ± 0.036[b]
474	0.704	2-Furfuryl methyl ether	13679-46-4	1246	1247 [121]	0.214 ± 0.019[a]	0.217 ± 0.01[a]	0.209 ± 0.018[a]	0.213 ± 0.017[a]
783	0.504	**Furfural**	98-01-1	1473	1460 [83]	52.667 ± 5.573[a]	46.062 ± 2.965[b]	49.165 ± 2.882[ab]	46.815 ± 4.157[b]
819	0.704	**Benzofuran**	271-89-6	1506	1489 [122]	1.644 ± 0.235[a]	1.281 ± 0.078[b]	1.188 ± 0.115[b]	1.618 ± 0.096[a]
831	0.536	**1-(2-Furanyl)-Ethanone**	1192-62-7	1513	1510 [121]	3.449 ± 0.205[a]	3.733 ± 0.253[a]	3.474 ± 0.249[a]	3.080 ± 0.141[b]
915	0.536	**5-Methyl-2-furfural**	620-02-0	1583	1578 [121]	9.412 ± 1.104[a]	8.889 ± 0.664[a]	8.781 ± 0.742[a]	4.893 ± 0.242[b]
930	0.760	**2-Methylbenzofuran**	4265-25-2	1596	1563 [120]	0.671 ± 0.081[a]	0.468 ± 0.045[b]	0.446 ± 0.037[b]	0.732 ± 0.049[a]
960	0.592	**2-Acetyl-5-methylfuran**	1193-79-9	1619	1608 [124]	0.078 ± 0.008[ac]	0.094 ± 0.011[b]	0.088 ± 0.013[ab]	0.074 ± 0.006[c]
969	0.624	Ethyl 2-Furoate	614-99-3	1629	1621 [82]	7.224 ± 0.798[a]	7.217 ± 0.455[a]	6.701 ± 0.441[a]	6.797 ± 0.341[a]
1017	0.440	2-Furanmethanol	98-00-0	1670	1669 [125]	14.885 ± 2.342[a]	13.991 ± 1.619[a]	13.485 ± 1.801[a]	13.181 ± 0.980[a]

1065	0.472	**Itaconic anhydride**	2170-03-8	1711	1680 [102]	0.284 ± 0.114[ab]	0.218 ± 0.034[a]	0.247 ± 0.050[a]	0.330 ± 0.042[b]
1113	0.456	2(5H)-Furanone	497-23-4	1756	1767 [126]	0.263 ± 0.045[a]	0.302 ± 0.054[a]	0.272 ± 0.041[a]	0.284 ± 0.046[a]
		Ketones							
		Aliphatics							
165	0.928	Pinacolin	75-97-8	997	978 [102]	0.131 ± 0.016[a]	0.120 ± 0.022[a]	0.126 ± 0.016[a]	0.132 ± 0.019[a]
234	0.648	2,3-Pentanedione	600-14-6	1068	1068 [53]	0.440 ± 0.048[a]	0.457 ± 0.058[a]	0.409 ± 0.027[a]	0.444 ± 0.032[a]
384	1.120	**2-Heptanone**	110-43-0	1186	1183 [156]	0.311 ± 0.039[a]	0.370 ± 0.030[b]	0.344 ± 0.042[ab]	0.204 ± 0.018[c]
489	1.280	**3-Octanone**	106-68-3	1257	1261 [127]	0.358 ± 0.043[a]	0.341 ± 0.033[ab]	0.335 ± 0.028[ab]	0.308 ± 0.023[b]
675	1.232	**2-Nonanone**	821-55-6	1390	1387 [82]	0.517 ± 0.074[a]	0.477 ± 0.034[a]	0.475 ± 0.031[a]	0.391 ± 0.024[b]
870	1.216	**3-Hexen-2-one**	763-93-9	1544	--	0.051 ± 0.006[a]	0.048 ± 0.004[a]	0.045 ± 0.005[a]	0.063 ± 0.004[b]
936	1.376	**2-Undecanone**	112-12-9	1599	1599 [128]	0.199 ± 0.044[a]	0.161 ± 0.016[b]	0.140 ± 0.024[c]	0.153 ± 0.015[bc]
		Cyclics							
564	1.320	2,2,6-Trimethylcyclohexanone	2408-37-9	1310	1312 [129]	0.529 ± 0.109[a]	0.432 ± 0.029[a]	0.454 ± 0.029[a]	0.463 ± 0.035[a]
927	0.472	**2-Cyclopentene-1,4-dione**	930-60-9	1590	1576 [130]	0.344 ± 0.040[ab]	0.375 ± 0.042[a]	0.352 ± 0.044[ab]	0.322 ± 0.024[b]
		Lactones							
1053	0.584	**γ-Caprolactone**	695-06-7	1703	1694 [147]	0.121 ± 0.010[a]	0.141 ± 0.020[b]	0.128 ± 0.017[ab]	0.122 ± 0.007[a]
1404	0.544	Dehydromevalonic lactone	2381-87-5	2015	--	0.042 ± 0.011[a]	0.047 ± 0.009[a]	0.043 ± 0.011[a]	0.042 ± 0.008[a]
1407	0.800	γ-Nonalactone	104-61-0	2024	1998 [113]	0.280 ± 0.072[a]	0.274 ± 0.030[a]	0.253 ± 0.036[a]	0.243 ± 0.019[a]
1416	0.464	Pantolactone	599-04-2	2031	2033 [131]	0.470 ± 0.107[a]	0.519 ± 0.104[a]	0.463 ± 0.164[a]	0.527 ± 0.096[a]
		Naphthalene compounds							
1089	0.760	**Naphthalene**	91-20-3	1734	1740 [103]	0.445 ± 0.268[a]	0.200 ± 0.018[a]	0.190 ± 0.020[b]	0.196 ± 0.013[b]
1245	0.776	**1-Methylnaphthalene**	90-12-0	1880	1875 [132]	0.073 ± 0.037[a]	0.030 ± 0.005[b]	0.030 ± 0.003[b]	0.059 ± 0.007[a]
1362	1.000	**1,8-Dimethylnaphthalene**	569-41-5	1989	1989 [133]	0.121 ± 0.024[a]	0.056 ± 0.008[b]	0.063 ± 0.009[c]	0.127 ± 0.019[a]
1551	0.704	**Cadelene**	483-78-3	2147	2200 [134]	0.187 ± 0.035[a]	0.091 ± 0.015[b]	0.108 ± 0.014[c]	0.281 ± 0.044[d]
		Norisoprenoids							
984	0.912	Safranal	116-26-7	1642	--	0.210 ± 0.021[a]	0.226 ± 0.013[a]	0.211 ± 0.014[a]	0.220 ± 0.010[a]
1176	1.088	**β-Damascenone**	23726-93-4	1814	1830 [53]	1.360 ± 0.363[ab]	1.213 ± 0.099[a]	1.148 ± 0.127[ab]	1.058 ± 0.061[b]
		Phenol-derivatives							
1227	0.520	**Guaiacol**	90-05-1	1862	1867 [53]	0.829 ± 0.164[ab]	0.812 ± 0.090[a]	0.739 ± 0.092[ab]	0.697 ± 0.044[b]
1326	0.576	**Creosol**	93-51-6	1952	1956 [136]	0.245 ± 0.043[a]	0.214 ± 0.021[ab]	0.202 ± 0.027[bc]	0.186 ± 0.013[c]
1395	0.416	Phenol	108-95-2	2007	2008 [53]	0.427 ± 0.100[a]	0.409 ± 0.058[a]	0.387 ± 0.046[a]	0.380 ± 0.059[a]
1395	0.448	2-Methylphenol	95-48-7	2013	2012 [94]	0.210 ± 0.050[a]	0.214 ± 0.036[a]	0.211 ± 0.022[a]	0.206 ± 0.025[a]

1419	0.624	4-Ethylguaiacol	2785-89-9	2034	2039 [83]	2.900 ± 0.781[a]	2.609 ± 0.293[a]	2.412 ± 0.298[a]	2.595 ± 0.179[a]
1488	0.576	2-Methoxy-4-propylphenol	2785-87-7	2094	2099 [157]	0.310 ± 0.092[a]	0.256 ± 0.030[a]	0.245 ± 0.032[a]	0.258 ± 0.021[a]
1593	0.496	**2,4-Di-tert-butylphenol**	96-76-4	2186	2280 [130]	0.601 ± 0.386[a]	0.439 ± 0.162[a]	0.358 ± 0.140[a]	0.236 ± 0.033[b]
		Sulphur compounds							
219	0.664	**Methylthiolacetate**	1534-08-3	1054	1057 [119]	0.504 ± 0.055[ab]	0.466 ± 0.032[a]	0.497 ± 0.028[ab]	0.529 ± 0.044[b]
243	0.752	**Dimethyl disulfide**	624-92-0	1074	1063 [137]	0.265 ± 0.076[a]	0.301 ± 0.074[ab]	0.283 ± 0.063[a]	0.364 ± 0.074[b]
849	0.632	**2-Methyl-3-thiolanone**	13679-85-1	1528	1525 [130]	0.372 ± 0.051[a]	0.482 ± 0.048[b]	0.426 ± 0.046[ab]	0.660 ± 0.065[c]
1047	0.528	**2-Thiophenecarboxaldehyde**	98-03-3	1695	1684 [81]	0.209 ± 0.014[a]	0.224 ± 0.019[a]	0.218 ± 0.020[a]	0.256 ± 0.016[b]
1074	0.480	**3-(Methylthio)-1-propanol**	505-10-2	1720	1719 [83]	7.511 ± 0.802[a]	8.410 ± 1.275[ab]	7.420 ± 1.209[a]	8.697 ± 0.957[b]
		Terpenic compounds							
396	2.192	**Limonene**	5989-54-8	1193	1191 [135]	5.386 ± 4.139[a]	1.362 ± 0.208[b]	1.238 ± 0.148[b]	1.650 ± 0.124[c]
402	2.352	**Eucalyptol**	470-82-6	1198	1195 [135]	0.307 ± 0.105[ab]	0.216 ± 0.022[b]	0.304 ± 0.072[a]	0.163 ± 0.085[b]
468	2.032	**γ-Terpinene**	99-85-4	1243	1274 [89]	0.558 ± 0.180[ac]	0.387 ± 0.042[ab]	0.360 ± 0.058[b]	0.634 ± 0.057[c]
516	2.024	**α-Terpinolene**	586-62-9	1276	1315 [89]	1.112 ± 0.789[a]	0.315 ± 0.031[b]	0.318 ± 0.036[b]	0.508 ± 0.025[a]
618	1.680	Rose oxide	16409-43-1	1349	1339 [138]	0.451 ± 0.053[a]	0.445 ± 0.038[a]	0.415 ± 0.026[a]	0.421 ± 0.019[a]
777	1.224	**Nerol oxide**	1786-08-9	1470	1470 [158]	5.395 ± 0.637[a]	5.118 ± 0.238[a]	4.768 ± 0.275[b]	5.443 ± 0.393[ab]
822	1.088	**Camphor**	76-22-2	1506	1458 [140]	0.122 ± 0.037	--#	--#	--#
876	0.832	**Linalool**	78-70-6	1553	1526 [135]	10.367 ± 1.532[a]	9.755 ± 0.561[a]	9.379 ± 0.601[ab]	8.952 ± 0.371[b]
936	0.936	**Terpinen-4-ol**	562-74-3	1601	1605 [101]	1.283 ± 0.181[ab]	1.330 ± 0.099[a]	1.229 ± 0.095[ab]	1.202 ± 0.070[b]
1047	0.808	α-Terpineol	98-55-5	1696	1686 [141]	5.406 ± 1.073[a]	5.479 ± 0.562[a]	5.053 ± 0.685[a]	5.169 ± 0.491[a]
1164	0.728	**Nerol**	106-25-2	1800	1803 [142]	0.089 ± 0.040[a]	0.084 ± 0.009[a]	0.069 ± 0.018[a]	0.050 ± 0.006[b]
1215	0.688	**Geraniol**	106-24-1	1851	1867 [89]	0.254 ± 0.055[ab]	0.274 ± 0.038[a]	0.253 ± 0.046[ab]	0.217 ± 0.018[b]
1434	1.048	**Nerolidol**	7212-44-4	2047	2046 [145]	0.520 ± 0.228[a]	0.290 ± 0.040[b]	0.315 ± 0.056[b]	0.401 ± 0.036[a]
		Other Compounds							
525	0.616	Acetic anhydride	108-24-7	1282	--	1.284 ± 0.148[a]	1.282 ± 0.195[a]	1.201 ± 0.185[a]	1.108 ± 0.139[a]
639	1.088	**Indane**	496-11-7	1364	1365 [103]	0.172 ± 0.058[a]	0.116 ± 0.009[b]	0.114 ± 0.012[b]	0.154 ± 0.011[a]
828	0.864	Ethyl sorbate	2396-84-1	1511	1501 [136]	0.136 ± 0.032[a]	0.140 ± 0.023[a]	0.128 ± 0.013[a]	0.135 ± 0.013[a]
942	0.568	**Benzonitrile**	100-47-0	1608	1583 [146]	0.191 ± 0.036[ab]	0.216 ± 0.028[a]	0.192 ± 0.021[a]	0.163 ± 0.013[b]
945	0.656	**1-Ethyl-2-formylpyrrole**	2167-14-8	1608	1610 [159]	0.266 ± 0.014[a]	0.304 ± 0.022[b]	0.277 ± 0.018[a]	0.349 ± 0.024[c]
963	0.584	**1-Methyl-2-formylpyrrole**	1192-58-1	1624	1620 [160]	0.190 ± 0.014[a]	0.213 ± 0.021[b]	0.197 ± 0.020[ab]	0.185 ± 0.010[a]
1158	0.528	**Tetrahydro-2H-pyran-2-one**	542-28-9	1797	1780 [101]	0.220 ± 0.036[ab]	0.253 ± 0.045[a]	0.218 ± 0.032[ab]	0.193 ± 0.012[b]

[a] Retention time for first (^1t_R) and second (^2t_R) dimensions in seconds. [b] RI: Linear Retention Index obtained through the modulated chromatogram. [c] RI: Linear Retention Index reported in the literature for DB-FFAP column or equivalents. The results are expressed as the averages of 4 bottles × 3 replicates ($n = 12$) ± the standard deviation. The concentrations are expressed as equivalents of 3-Octanol. Different superscript lowercase letters in a row represent statistically significant differences between wines bottled with different stoppers at $p < 0.05$, using Two-Way ANOVA (followed by Tukey´s multiple comparison test) in GraphPad prism. # – not detected.
